# Drivers and drainers of compassion in intensive care medicine: An empirical study using video vignettes

**DOI:** 10.1371/journal.pone.0283302

**Published:** 2023-03-23

**Authors:** Shahla Siddiqui, Christiane Hartog

**Affiliations:** 1 Department of Anesthesia, Critical Care and Pain Medicine, Beth Israel Deaconess Medical Center, Harvard Medical School, Boston, MA, United States of America; 2 Department of Anesthesiology and Intensive Care Medicine, Charité Universitätsmedizin Berlin, Berlin, Germany; University Medical Centre Ljubljana (UMCL) / Faculty of Medicine, University Ljubljana (FM,UL), SLOVENIA

## Abstract

**Background:**

The aim was to determine what factors drive and enhance compassionate care behaviors in the ICU setting and which factors drain and negate such caring attitudes and behaviors.

**Methods:**

Qualitative, focus group discussions using video vignettes. 20 participants agreed to be part of 3 separate focus groups facilitated by the authors.

**Results:**

Thematic analysis revealed emphasis on behavior and nonverbal cues, clinical decision making, communication and sensitivity, and building humane relations. The results show that physicians feel driven by the humanity and sensitivity felt in ICU work, however, there exists structural incompetence, as well as the stress and personal -systemic imbalances of ICU work, which leads to burnout and erosion of such motivations, draining compassion.

**Conclusions:**

Regulatory and scheduling practices must be examined to foster the growth of compassionate behaviors and attitudes in healthcare, and these should be treated as essential patient centered metrics.

## Introduction

Studies have shown that despite advances in patient centered outcomes deficiencies remain in delivering compassionate healthcare [1]. In the US a large proportion of the public believes that the healthcare system is not compassionate [2]. Evidence also points to the fact that physicians miss up to 90% of opportunities to respond to patients with compassion [3]. The ICU is a setting where life and death decisions are made and sensitivity and compassion to the patient and family suffering are most needed. Since doctors enter the demanding field of medicine because they wish to help people and relieve suffering why is there scarcity of compassion in healthcare [4]?

The normative premise for healthcare is that physicians and organizations have a moral responsibility to dispense proper care and act on the basis of particular responsibilities towards their patients [5]. This concept of caring is an essential component of the complex entity of compassion, which in healthcare underpins the relief of suffering and feeling of empathy and connection with other sentient beings [6]. The intention and focus of care and compassion are “*sensitivity to suffering in self and others with a commitment to try to alleviate and prevent it*” [7]. This concept and its benefits have been recognized in medical as well as spiritual tradition over centuries [8]. In the intensive care unit (ICU), critically ill patients are cared for by a team of physicians and nurses. Recently, the study of compassion has revealed many levels of positive effects on clinical and psychological wellbeing, as well as prosocial and caring behaviors [9].

The emotional burden of suffering in the ICU (of the patients’, as well as their loved ones’) invokes feelings of duty, caring, empathy and compassion which encapsulates the physicians’ role as an altruistic and kind responder motivated by not just humanistic feelings but also the moral ontology of a virtuous physician-healer [10]. Compassionate and caring behaviors result in better outcomes for patients and their families [11]. However, there is an intersection of compassionate care in the ICU, and the complexities surrounding communication, decision making, the role of families as surrogates and complex interpersonal relationships, which disrupts the balance of this moral premise and raises tensions. Studies done on physician compassion from a patient perspective emphasizes the focus on listening and presence, or awareness of the patient’s emotional state [12]. This not only builds trust within the patient physician relationship, but compassionate care also enhances resilience amongst physicians and prevents burnout [13].

To understand how physicians working in the ICU construct their perception of compassionate care we hypothesized that showing scripted video case vignettes with different themes around compassionate care in the ICU can elicit responses that will help achieve this goal. Our aim was to describe compassionate behaviors in the ICU and study factors that enhance and those that drain such behaviors, with an aim to enable recommendations for practice and training.

## Methods

This was an observational, qualitative study using video case vignettes and focus group discussions. The Standards for reporting qualitative reports guidelines were used [14]. Participants were invited via email of 2 international critical societies. Informed consent was obtained via email prior to the focus group discussions. Two scripted video vignettes were shown to 3 groups of participants of virtual focus group discussions. These video scenarios were 5 minutes each and were developed using real life scenarios occurring in the ICU. The 2 videos chronicled interactions around the end-of-life care of an African American patient who had expressed previously the desire not to receive non-beneficial life prolonging measures. The ICU attending (male), bedside nurse (female), and a junior (female) resident discussed goals of care which included aggressive measures or goal concordant and compassionate care. In one video the attending was portrayed as compassionate, inclusive, and kind, and conversely in the second video he was shown as distracted, dismissive, and did not appear to care about the patient’s wishes and preferences but wanted to ‘make a bed’ in the ICU. (S1, S2 and S3 Appendices and S1 and S2 videos labeled Compassion and No Compassion using nurse and physician volunteer actors).

These contrasting videos were followed by a general discussion amongst 25 participants in three divided focus groups conducted among participants from different parts of the world and who were members of the *European society of intensive care medicine (ESICM)* and the *Society of critical care medicine (SCCM)*. Structured prompts were used by the principal investigator who moderated these discussions to elicit responses about compassionate behaviors (or the lack of it) in the ICU. The videos were used to bring out salient aspects of compassionate care and resonate with the participants as examples of the display or the lack of compassion and empathy. Transcripts of all three focus groups were analyzed by thematic analysis and codes were iteratively developed by the study team. Codes were compared and any disagreements were resolved by mutual discussion amongst the study team. Institutional review board approval (2021P000065) was obtained at Beth Israel Deaconess Medical Center, Boston, for this study and participants were invited from the 2 ICU societies online. The virtual focus groups were conducted over an online platform (Zoom®). Most of the participants were physicians, with 1 ICU nurse.

## Patient and public involvement

No patient was involved.

## Results

20 participants responded to the invitation and 3 separate focus groups were conducted by the principal investigator. After viewing the videos participants agreed that the scenarios resonated with them, and they could recollect encounters whilst working in the ICU like the 2 situations. Both the verbal and non-verbal communication was apparent to the participants, and they reiterated the value of the underlying factors that may lead to such behaviors. These included discussion on the following aspects of the 2 scenarios.

### Behavior and nonverbal cues

In the situation where the ICU attending displayed unfavorable behavior, the participants quickly voiced their disgust and believed it too ‘terrible to watch’. They felt the contrast was striking between the two scenarios with the second one showing a softer and kinder encounter, whilst the first one was unpleasant where the dominating male attending was insensitive, condescending, and dismissive. The participants of the focus groups discussed the emotions they felt watching these scenarios play out and related to the real-life issues they themselves encountered in ICU care. They could pick up on the inattentive, arrogant stance of the attending and the clear lack of compassion in the second video. However, as one participant stated, “it is easier to see lack of compassion” when the interaction occurs in front of us. It invokes feelings of distrust, anger, hurt and frustration. The vignette where compassion was displayed in a kind and gentle way evoked feelings of calm, trust and confidence in the intent of the team involved. Participants reflected on how patients and families probably feel similar emotions when faced with similar situations.

Some described the non-verbal communication:

“Something I always have the feeling that when you communicate to a patient in the intensive care unit, your own body language is very important. For example, I would never stand next to a patient when talking to him, I would always sit next to the patients, next to the bed myself. So, the language and actions are very important to show your compassion.”

### Clinical decision making

Participants vocalized that these decisions happen often in the ICU and considerations of resource allocation are ever present. They noted that in the first scenario the attending physician showed a dismissive attitude towards the wishes of the patient and consideration for their family, which they found abhorrent. They pointed out that it is often easier to define compassionate behavior when it is missing. In the second scenario, they noticed the inclusive, thoughtful consideration given to not only the patient but also the team members. Such an example of clinical decision making establishes a connection with the suffering of the patient and enhances good and ethical practices. Some felt that decisions and behaviors are contextual and change with circumstances:

“….it’s not a fixed issue, very dynamic, same physician behave differently at different points, and even for the same patient can behave different. Compassion may be very good in the beginning but later on maybe not, because of various factors. So, this is a very important to understand, and there are also the relatives, they also behave so it’s very intermingled dynamic situations.”

### Communication and sensitivity

Participants were aggravated by the audacity of the first video and recognized parallels in their own environment. The video produced flashbacks for many participants. They reiterated other similar scenarios in their ICUs usually around end-of-life care or communication. They voiced that caring and compassion seem to stem from sensitivity and emotional resonance, which comes from being present in the situation. Participants felt that showing their compassion is linked to being sensitive to the emotions of their patients and their families. This ‘art’ of communication is often vital in forming trust and laying the foundations of a healthy patient -physician relationship. They expressed their opinions about how compassion can be expressed by good communication and can be taught to some degree by reflection and self-awareness around attitudes. The hurdles faced in delivering compassionate care are usually around lack of self-awareness and lack of sensitivity.

“One is compassion and the other is communication, appropriate and good communication. And I think that what we saw in this video firsthand from my view was an example of really bad communication, and rude, communication, and maybe an example of very much better communication.”

### Building humane relations

Most clinicians felt the moral imperative to ‘do good’, build humane relations and make the correct decisions. However, they understood that there were hurdles in delivering such care. As one physician said:

“It has definitely been a struggle to remain compassionate when you are running around with your hair on fire all the time!!”

Participants reiterated that the healthcare system, with its inherent transactional demands, undermines ideals and leads to alienation and burnout. For example, physicians feel that instead of spending time forming important humane connections with patients and families they need to pay attention to the electronic health record and its multitude of documentation. An important negative example is when physicians focus on physiological abnormalities instead of seeing and relating to the whole person and the nuances of the care decisions in their trajectories. For example, the impact of decisions made in the care of an ICU patient can influence, not just the immediate outcome of the patient but also far-reaching consequences down the line. Participants drew parallels of the videos with their own practice experiences. Some voiced their intention to always have a broader approach to patient care incorporating humanism, respect and to maintain a positive engagement with their patients and teams.

“I would reflect on the interpretation and context of both videos. They presented data points that were the same but the teams interpreted them differently and from a context standpoint you saw in the first video, singular focus on very specific items that may or may not be correctable and in the second video, you saw, I think, a much broader approach to the patient’s care including thinking about the trajectory and what that meant for what care was possible.”

To others compassionate care meant more than communication with words:

“When I think of compassion and what matters to families, it definitely has something to do with good communication, but it’s not only that. Although I’m not very strong believer but I grew up in this way and I remember the story of Job in the First Testament first book of the Bible, and Job lost everything. And then he sat in the ashes of his house and tried to console himself and what happened was that friends and family gathered around him and just sat down with him. And for me, this is one of the strongest metaphors for compassion, just to sit with someone in his ashes.”

“Sitting with someone in the ashes” embodies relation-building, not taking the physician-centered approach, not offering (unwanted) solutions or advice, but taking the other persons’ view and being at their side.

### Reflection about obstacles to compassionate care and training

The participants reflected on other aspects of their care and behaviors after discussing the case vignettes. They drew upon the emotions they felt when watching the videos.

However, they also noted that they have changed, or been made to change, in order to be more ‘efficient’. Many lamented that the idealism they felt on joining medical training and on first becoming physicians had changed over time leading to burnout. They stated that emotional resonance ‘took time’ and often they did not have that. Some also reflected on the need for cultural and gender appropriate behavior training to eliminate unintentional or unnoticed microaggressions, as displayed in the first video. They agreed that compassionate care required an element of emotional intelligence without emotional involvement, something which can be taught and learnt. One participant stated it this way:

“There are things better caught than taught, and I think compassion tends to fall within that category, but it doesn’t mean that you can’t break it down into components and talking about things that are important, like presence, like establishing some sort of a connection. So, the short answer is no, I think it’s probably better to remodel it but the long answer is we can probably provide material that prepares a learner to absorb as much of the experience as possible.”

From this discourse, the perspectives of the following entities involved in these scenarios are displayed.

#### The team

In the ICU the multi-disciplinary team brings a three-dimensional perspective, with nurses, physicians and trainee physicians involved in decision making. Compassionate behavior is expected from all team members to encapsulate a satisfying patient and family experience. It also brings satisfaction and fulfilment to clinicians, which was part of the moral imperative, the ubiquitous intention and the dismay expressed when watching the negative example in the first video. Good communication, sensitivity and thoughtful consideration was stressed as part of compassionate care. This was evident in the second video which resonated with participants.

#### The individual

Participants reflected on their work and emotions after watching the videos. They felt hatred and abhorrence at the video that lacked compassion, and they appreciated the humanity and dignity displayed in the second video. Participants agreed that individual compassionate behavior in the ICU is linked to building a humane relation with the person of the patient but is also often reflective of considerate and thoughtful relationships with members of the multi-disciplinary teams.

#### The system

Participants recognized limitations to their expression of compassionate behaviors and found these to be linked at times to the healthcare system requirements. Capacity strain, time pressures and burnout were factors identified by participants that hampered compassionate behaviors.

#### The patient and their family

These are the center of care in the ICU. Literature has highlighted how families perceive the care they and their loved ones receive in the ICU and the gaps in communication and compassion this has identified. Our videos identified some aspects of attitudes and behaviors displayed in the ICU by the healthcare team (which were recognized as often displayed in real life by the participants), and which lacked compassion and respect.

The burnout felt by ICU staff has been unprecedented, but it is underscored by the barriers to compassionate care that exist in our healthcare systems which impede the natural desires of physicians to act compassionately. This compassion fatigue in itself can cause moral distress. As one participant shared:

“I think it really impacts care, I am burned out I am fatigued, and I remember a patient who was still on High Flow for three weeks, very comfortably. And in the end, you know, it was a 50,60 something year-old, next-door neighbor kind of person, and he had decided to make himself DNR. And I remember, you know, in the last 24 hours when I knew he was going to pass, and the wife had made the decision to move towards comfort instead of just aggressive care with DNR DNI. The nurses were tearing up, and I wasn’t, and that wasn’t me and you know, I thought, oh, my gosh, what has happened to me, you know, and I felt really guilty. You know, that I couldn’t make that eye contact, that I couldn’t sit with the family. I did, you know, but not the way I would normally sit with them in their uncomfortableness. And I was grateful later that night when I went home and I cried because then I realized, okay, I’m still human. I still can be compassionate.”

## Discussion

This study was aimed at eliciting the various aspects and understanding of compassionate care in the ICU environment and identifying some key hindrances to such care. This study revealed that ICU physicians show intention and believe the moral imperative for compassionate care, however through communication constraints, structural incompetent expectations, and environments (for example, electronic health record demands superseding time spent with a dying patient, or family separation at the time of death during the pandemic), work strain burdens and burnout, these compassionate intentions are drained. These hurdles are manifest in their decisions and clinical choices, interpersonal connection or lack of it, and nonverbal behaviors.

Compassionate care should, therefore, encompass good communication, sensitivity, thoughtful consideration of patients and co-workers and having a humane relation with the patient and involved staff. This study also shows that although ICU physicians share a moral imperative and intention for compassionate care and feel relief and confirmation of their values and convictions in the video depicting compassion. They also relate to the audiovisual depiction of the unpleasant lack of insensitivity and compassionate care shown in the negative video.

The recent COVID-19 pandemic had highlighted existing structural deficits with resulting lack of work life balance which produced unprecedented levels of burnout and indeed unmasking of various levels of stress borne by ICU staff [15]. “This is coupled now in the era of electronic health records, where there are rigorous data to show that health care providers spend more time looking into computer screens than looking their patients in the eyes” [16]. Recent focus on systemic racial and ethnic biases are also embedded in these structural developments in healthcare norms. Yet the role of such structural forces on health and healthcare, which arise from social, economic, and political factors are drivers of poor health outcomes [17]. These disparities also increase the burden on healthcare workers who manage these outcomes [18]. In summary, many aspects of ICU care can be the *drivers and drainers* of compassionate care. This is similar to findings by critical care nurses who have described that unmet expectations and demands from management and colleagues are hindrances while provision of patient-centred care enhances their capacity for compassion. The pandemic has affected everyone in a negative way, not the least ICU physicians and has magnified existing structural deficits. Structural competency, (i.e. understanding which structures impede or drive the delivery of optimal care), can contribute to understanding one’s own compassion or lack thereof [19]. The current moment in healthcare with this increased awareness of healthcare burdens and structural incompetence, can provide an unprecedented opportunity to shift the working environment and training to comply with the patient and provider demand for compassionate care [20]. This can enhance outcomes for patients and families as well as provide a more satisfying and fulfilling experience for physicians and nurses. In this digital world, one of the key findings is how physicians feel that instead of spending time forming important humane connections with patients and families they need to pay attention to the electronic health record and documentation.

In his book ‘The soul of care: the moral education of a husband and a doctor’ (2019) Professor Kleinman writes “Caregiving is long, hard, unglamorous work—at moments joyous, more often tedious, sometimes agonizing, but it is always rich in meaning [21].” The results of this study resonate with Professor Kleinman’s conclusions that physicians connect with the practical, emotional and moral aspects of caregiving.

Our study had the inherent limitations of a qualitative study which was based on video case vignettes to invoke audio-visual cues for focus group discussions among intensivists from across the world. The discussion was based on reflections of participants and not on real life actions. Qualitative thematic analysis is subject to bias of interpretation [22]. This was mitigated by iterative discussion, constant comparison and coding and recoding. The videos were found to be powerful for eliciting responses, as one participant stated:

“A lot of learning opportunities in the first video, I think, you know, besides addressing the patient’s pain, which I think is also a component of compassion care, I think you guys hit the nail on the head about nonverbal communication. I think when you don’t address, you know, the issues in the room, it makes everybody really uncomfortable. And I think that effect trickles down to patient care. Eye contact is so important. Not just to the staff members, but you know, one of the things I’ve realized when you are sitting with a patient or family really like really connecting with that. It has actually for me, it’s a lot of mental energy, but it’s so powerful.”

Healthy and supportive work environments are imperative to the health and wellbeing of ICU staff as well as patient outcomes [23]. Logistic and systemic changes in the healthcare environment can improve wellbeing and satisfaction. A thorough exploration of emotional distress in relation to communication skills, ethical rounds, and mindfulness might provide an appropriate starting point for the development of further preventive strategies and development of compassionate behaviors. We also can foster compassionate organizational cultures by recognizing the needs of ICU physicians when delivering humanistic care and build on the findings of this study [24]. Such examples would include explicit teaching tools for trainees, clinical programs that encourage acts of compassion, counselling sessions and other interventions focused on enhancing connections with patients and families rather than electronic health records.

## Conclusion

It seems that physicians working in extremely stressful conditions share a moral imperative and are inspired by the humanism and compassion that fueled their initial motivation to join physically and mentally demanding healthcare work. However, these sentiments are eroded by the pressures of capacity strain, lack of staff, lack of compassionate skills training, dreariness and perfunctory electronic health record maintenance, and other non-humanistic rituals in healthcare, as these take up the precious time and energy rather spent in forming compassionate connections with patients and their families at times when these actions are most required.

## Supporting information

S1 Table Main points of analysis. (DOCX)

S1 Appendix (DOCX)

S2 Appendix (DOCX)

S3 Appendix (DOCX)

S1 Video Compassion video. (MP4)

S2 Video No Compassion video. (MP4)
