# Supplementary material for: Drivers and drainers of compassion in intensive care medicine: An empirical study using video vignettes
Source: PLoS One. 2023 Mar 23;18(3):e0283302. doi: 10.1371/journal.pone.0283302 (PMC10035878; doi:10.1371/journal.pone.0283302)
Supplement: S1 Table — (DOCX) [file pone.0283302.s001.docx]

| **Domains** | **Behavior and Nonverbal cues** | **Clinical decision making** | **Communication and sensitivity** | **Building humane relations** |
| --- | --- | --- | --- | --- |
| **ICU Team** | Body language | Consideration for family opinions | Emotional intelligence and presence | Avoiding dismissive behaviors |
| **Individual** | Emotions | Recognizing biases | Building trust | Feeling the pain of others |
| **System** | Time and burdens | Data and evidence | Allowing space and time | Avoiding jargon |
| **Patient and family** | Empathy and understanding of perspective | Shared decisions and control | Sharing vulnerability | Having access to the team and information |

**Table 1. Main points of analysis**
