# Supplementary material for: Drivers and drainers of compassion in intensive care medicine: An empirical study using video vignettes
Source: PLoS One. 2023 Mar 23;18(3):e0283302. doi: 10.1371/journal.pone.0283302 (PMC10035878; doi:10.1371/journal.pone.0283302)
Supplement: S1 Appendix — (DOCX) [file pone.0283302.s002.docx]

# Compassionate Care FGD 24^th^:

**9 Participants Including the PI Conducted through Zoom**

PI: As we all know the background of this study, we're trying to understand the, the construct around compassionate care in the ICU, and all of you have in some way, some connection to the intensive care unit, and the ethics around it. So if you don't mind, we'll just go around and have everyone just say their name which country they belong to, where they are right now, so that we can go on and then I will proceed with showing two very short videos that are homemade here~~,~~ and excuse the direction I was the director of those videos but I director in charge of that center simulation center was actually one of the leads in the movie. So, enjoy the movies and then we will have a set of some questions.

Speaker 1:Yeah, I'm R, and a retired sort of half and half type

PI: C

Speaker 2: Yes, hi my name is C I'm from and a physician actually GP by training, but I'm working in clinical ethics now, and healthcare researcher, C

Speaker 3: M I'm in, and I run the critical care units here.

PI : Thank you, Dr. K

Speaker 4:Yeah, I am intensivist, professor in from. Also, I am a member of ethics institutional ethics committee also.

PI: Great, thank you for joining us. Dr V

Speaker 5: Yes, my name is B triggers I've been intensively since about 25 years working in the Netherlands. And I did a surgical rotation in in the B with Dr. W silent. So, I know that hospital quite well.

PI: Excellent. Dr. M

Speaker 6: Yeah, please excuse my prediction of being late, but I had it in my mind for tomorrow.

PI: Have you joined us, please, if you could just introduce yourself

Speaker 6: S physiologists and intensive care intensivist from.

PI: Great. We have a group of your people here, we have an advantage. S would you like to introduce yourself.

Speaker 7:

Hello everyone. My name is S. I'm the program manager at which is in, the work of the center is focused on exploring consciousness and cognitive science.

Speaker 2: your sound is not so good, it's.

PI: She's saying that she's part of the, the study as part of the Center, and this is at. This is a new initiative where we are trying to understand constructs of consciousness and compassion, and a lot of neurocognitive work so we have some interesting and exciting, innovative studies going on. So I'm going to start with a video .

Speaker 6: I can hear no sound. I'm sorry. Speaker 5 : And that neither can I. Speaker 7:

I think we can do un-carrier thing to make, and then click un-share again before you share this on

the lower side of your screen and you can click here. And you're also muted. Okay. PI: So, I'm sharing now share the screen.

Speaker 7: Okay. On the lower left-hand corner.

PI: Okay, I will start from the beginning I'm sorry I'm new at this. I had hope you can hear

# 1^ST^ Video Playing:

Actor 1: for a vasopressors. Actor 2: It's at 2.4.

Actor 3 : I think we need to have surgery or I take a look at her. We got to get the bleeding under control.

Actor 1:Dr. R, she's post cardiac arrest and on for maximum dose basis, Actor 2: and a pH of 6.8 shootout

Actor 3: she needs to go to the OR to control bleeding,

Actor 2: her wishes were no heroic measures.

Actor 3: She was not aware of what she was saying the besides, we can't keep her in the ICU, we need the bed. She

Actor 2: should we call her husband, or son at least, and have them come and sit with her. Her stomach is really distended and she's in pain could begin to some morphine.

Actor 3: morphine, it's just going to kill her. We need the bed we need to get her out of here. PI: Okay, I'm going to share another video now. Let me see if this is share some

# 2^nd^ Video Playing:

Actor 1: original press vasopressin,

Actor 2: vasopressin is at 2.4, and a pH of 6.8. Actor 3: We know what our wishes were?

Actor 2: She doesn't want to have any heroic measures. Actor 3: When we talked with family.

Actor 1: I think we should call her husband.

Actor 2: You know I can get hold of her husband and her son and have them come by. Do you think we could start some morphine, she said, Her stomach's distended in a lot of pain.

Actor 3: I think so we have to be really careful about the dosage

Actor 2: certainly, certainly, I do. I can also call on social worker and the palliative care, just to be available as well.

Actor 3: I think that's a good plan and what do you think? Actor 2: I agree I think that's a great plan.

PI: Okay, so I'm going to stop sharing now. And so those are two very short videos. They may be a little naive in the way that they presented compassion and then no compassion in the first video and then compassion in the second video, but it's just an icebreaker to bring out some important points that can occur at times, most people are not very obvious in the way that they behave in the ICU however there's certain subtleties and nuances that do come out, which have brought up dissatisfaction and moral distress amongst the members of patients and families. So, if we can start with going around the table and just asking people what they thought of the two videos and

their thoughts on compassion and then we got a set of some structured questions which I can go through, but just whatever your thoughts are. Can we start, let's just go down the list? Dr. V, it's your first on my list of participants.

Speaker 5: Well, isn't that even artificial, but it was obvious that the first video is heartbreaking and terrible to watch, and I think the way that people look at palliative care that they need a lot to learn. The second video is a little bit more. Well, I can recognize the way they talk. On the other hand, I would never have such a discussion at the bedside. That makes it a bit awkward, but it might be my personal experience when, when a person doesn't want a certain amount of care isn't pain, then it's obvious that that pain treatment is and palliative care is the main goal of care, and we should do that as, as consistent as possible.

PI: Thank you, Dr. M

Speaker 6: Yes, I think these are very short clips and very condensed in a way so maybe my thoughts with this would maybe to kind of RK types of dealing with a certain situation. But nonetheless, we just tried not to take too much word by word. What happened, I can find, I can, I can remember, sort of these discussions in both ways in our institution. So, in a way it was maybe naive but, in a way, it reflects what happens every day. This when I first thought about this.

PI:

Okay, we'll get into detail later and I have some leading questions to bring more out from the deep depths of your memories as well.

Speaker 2:

Yes, I agree with what has been said these are two condensed typical situations which, which in our hospital, in our part of the world you're going, you can find them both. And the third alternative would be from the view of Netherlands where the palliative care is instituted automatically without discussing it first with maybe the physician is in charge, so I see these are three possible trajectories. The third not being shown but having been talked about

PI: sure, any different thoughts R? Speaker 1:

well I suppose my initial thoughts are, what a nasty work in first video, the attending is presented

with, a nasty presentation that will be nasty to the people around him both the patient and the colleagues, so it comes across very vividly. And in the second we get a much more cooperative sort of atmosphere that's my initial thought

PI: M you are next in my list, Speaker 3:

I would, I would reflect on focus interpretation and context both videos presented data points that were the same with the teams interpreted them differently and from a context standpoint you saw in the first video, singular focus on very specific items that may or may not be correctable and in

the second video, You saw I think a much broader approach to the patient's care including thinking about the trajectory what that meant for what care was possible.

PI: That's very interesting. I'm going to pick on you for that. Thank you. Could you unmute Dr G

you're muted.

Speaker 4: Sorry. Yep. So the presenters in urine was a post cardiac arrest, extreme the pH, and the height, but to talk about that palliative care at this point of time we don't know the background because the scenario, which created this extreme physiological extreme pathophysiological condition can be reversible also, as well as the team, the members who are experienced or not experienced and also important to decide to think about the palliative versus intensive care at this point of time, so those things also to be considered while peace are encouraged, talking with our trainees, basically. So many times, patient get revived with cardiac arrest with a pH 6.8 and hydrocephalus.

PI:

great. Thank you so much for your thoughts. So just going back to the left anyone else no I had everybody here. Thank you for your initial thoughts on that very crude video, but I really wanted to bring out the aspects of compassion versus the understanding of no compassion and as some of you said that it was really the body language and looking at broader aspects and the patient's preferences that came out. So, do you think that compassionate care. Basically, embodies looking at underlying issues rather than the actual objective data, do you think you have any thoughts on that. Anyone can speak up now. If you have any thoughts on what compassionate care means and how our initial problem is that we don't know the definition of compassionate care. And then how can we teach this to our trainees if it's something very nebulous and abstract, how do you, did you see that the body language of the attending in the second video was, he was holding his hands and he was asking for opinions from his team members, he was not very sexist, not very rude. Whereas in the first video he had his arms folded and this was all from the director who was acting as the attending he the body language was very different words he chose the inclusiveness of the female trainee and the nurse. Everything was incorporated in that how do you define that. Any thoughts on that. M, do you spoke something about looking at things from different lenses.

Speaker 3:

So if I would try to take what you just described and reconcile it with what I described, you know, A couple of things occurred to me one is taking the clinical situation and trying to map it to a problem that is solvable and looking at that problem through a multitude of lenses and so maybe one of the things that might be helpful here when we talk about a term like compassionate thinking about how to drill down and what it truly means is the perspective around which the goals of care are discussed in the beginning, thinking, I've selected myself because I mentioned that there's this bigger picture, whereas, focusing on individual correctable or not correctable details, doesn't take into account the clinical situation. So, putting that all together. Is there a way to describe a process where a clinician can map, their discussions and their prioritization of goals around socially sanctioned or acceptable norms of appropriate care humanity, dignity, professional interaction, those sorts of things

PI: Robin, do you have any thoughts on how we can pick on the sort of sort of abstract aspects of compassion in the delivery of care.

Speaker 1:

Well I think it depends to some except whether one's seeing it as a virtue, a personal characteristic. I tend to do that. And in those contexts, I think we're going to have compassion in all sorts of different circumstances and with different solutions to problems. So, we don't have to have to be on one side of a moral dilemma or another in order to be compassionate. And I suppose one can urge that when is always compassionate as a doctor, whatever side of the problems, best solutions will be made by his own.

PI:

Interesting. Would you say that it doesn't matter what the side would be, that means, whatever the outcomes may be, you will still be compassionate.

Speaker 1: I would say yes, of course, it may be that some results will be very hard to bear. But nonetheless, they can be presented by a compassionate person in a better way than by a harsh non compassionate person.

PI: I see a hand up by Dr. M

Speaker 6: I was just thinking about two different things. One is compassion and the other is communication, appropriate and good communication. And I think that what we saw in this video firsthand from my view is was an example of really bad communication, and rude, communication, and maybe an example of very much better communication, but that might do that doesn't mean necessarily that we have that we can discuss the amount of compassion in it, so I will put up a pieces you can have a perfect good skilled communication, but you can also show no compassion at all.

PI: Interesting. Any other thoughts on communication as a separate from compassion,

Speaker 5: if I may say something I always have the feeling that when you communicate to a patient in the intensive care unit, your own body language is very important for example I would never stand next to a patient talking to him, I would always sit next to the patients, next to the bed and break myself. So, the language is very important to show your show compassion. And I would also like when you have such a conversation at the bedside, that you are better informed of the patients before you enter the room, because there was a lot of conversation about wishes which can be done outside of the patient room already so you're betting for me to start talking to the patient or talking over the patient,

PI: C you have your hand up

Speaker 2: way this is, this is going now kind of resonates with me because I keep thinking there's so many different ways of compassion, of course the first video, the attending was, was not a nice person, but we do not know whether he does not have his own way of compassion. I mean people are different and have different values and beliefs and sometimes compassion can

take the way like we do all we can and then it's over, which would not be maybe our compassion or compassion of many of us but, is it not a compassion. Compassion in the sense of putting yourself into the shoes of another and doing to them that you would like to have done to yourself. So, being compassionate myself I find it difficult to kind of place the label on the physician that he's not compassionate. He certainly didn't communicate well he's rude I would not, I mean he's, he's not a nice doesn't have a nice way of relating but I'm not sure whether he's not compassionate.

PI: Very interesting. so that means having the interpersonal skills which are not attractive or not pleasant may not mean that you are not compassionate. Very often the families consider physicians, non-compassionate or are they struck by compassion, and this comes out in complaints or it comes out in compliments that we get in the ICU. How do families differentiate compassionate care? Does anyone have any thoughts on the perception in my master's I did many focus groups with family members and even with excellent care it was some, some situations were considered lacking compassion by physicians. I'm also in a bereavement group recently where these are non-doctors non physicians who've lost parents during COVID, and they are all almost 90%. They think that the compassion is lacking amongst physicians, and it's it strikes me as surprising because we we've sort of are proud of how physicians have managed care during the pandemic. So, any thoughts on compassionate care versus how families perceive compassion.

Speaker 5: Well, let me say something, for example in the COVID crisis, there was a lot of problems, how you could communicate in a safe way to patients. And I really have always done my best, never to talk to the family, for example, in the room was a patient surrounded by machines, surrounded by machines, surrounded by PEEPS and surrounded by masks, I would always take them to a side room at two meters distance, and would take off my mask and communicate in a normal way. And that's what I do already. Since several months. At the very beginning of the crisis when everything was unknown. Then I would certainly mask myself and be more careful. But these last months, I've always had have had a priority to have a meaningful conversation in a compassionate way, which means that you're sitting in a quiet room. Show your face show your emotions, and I think that's what really was prompt from a family member that doctors were talking to them with their full masks and gowns and everything, and then it's very difficult to show your compassion.

PI: Thank you. Dr.G Can I ask you, do you think that there are any differences in how doctors communicate across the world in different situations, since you're in a situation which is very different to probably the ICU said we work in here. Yes,

Speaker 4: so the two important points has been raised in discussion currently it's the communities and the compassion, and this is very much immediate as the same person, same physician changes the composition and the communication for the same patient for different time, communication, as well as for the different patients maybe deal differently because it's one stressor also you had less time to talk, it's the various factors which influences actually compassion, it's not a fixed issue very dynamic, same physician behave differently at different points, and even for the same patient can behave different. compassion may be very good in the beginning but later on maybe not, because of various factors. So, this is a very important to

understand, and there are also the relatives, they also behave so it's very intermingled dynamic situations. The relatives, when they see some improvement is ongoing, they feel just everything is okay the doctor's opinion is very compassionate for the chaos. But there's a new complication new deterioration happened and that the same time, it's a piece and had less time to talk, or they are very stressful conditions, so maybe they are they have different processes for their particular situation. So, it also it changes, activity, that's it, that's my thought here,

PI: Thank you Dr. M you have your hand up.

Speaker 6: Yes. When I think of compassion and what matters, maybe to families. It definitely is, has something to do with good communication, but it's not only that, and I would. When I think about this, I am. I come from a Christian background, Although I'm not very strong believer but I grew up in this way and I remember the story of jobs in the First Testament first book of the Bible, and the Job lost everything. And then he sat in the ashes of his house, and tried to console his wounds and what happened was that friends and family gathered around him and just sit down with him. And for me, this is one of the strongest metaphors for compassion, just to sit with someone in his ashes. And when we think when I think about compassion. And what makes it up to a to a patient or a family. I think one thing could be to communicate what has happened. And the other thing is maybe just to sit in this picture to sit in the ashes, for me this is the point of compassion.

PI: Thank you. I think you're describing presence. yes, yes,

Speaker 6: this was a long story and then you just made it one word.

PI: It's something that I've been studying as well and there's a very good book about that as well, I'll share with you an email, M, do you think you've been in and it was horrible, we've all read and heard about how bad it was in the beginning and the surge. You think we can teach our trainees compassion through our experiences or do we role model it and they just have to learn.

Speaker 3:

There are things better caught than taught, and I think compassion tends to fall within that category, but it doesn't mean that you can't break it down into components and talking about things that are important, like presence like establishing some sort of a connection. So, the short answer is no, I think it's probably better to remodel it but the long answer is we can probably provide material that prepares a learner to absorb as much of the experience as possible.

PI: Right. And then of course it depends on the person's emotional intelligence as well whether they are open to learning or not. There's a very famous saying that when the learner is ready, the teacher will appear. So, if the student is not ready, you can role model all you want. They won't. They won't recognize what your role modeling, but you know some people are unteachable and some are very teachable, I find. So, I have a set of questions which my team is made and I have to go through them, I'll throw them out. Please, if you have a comment on that, that'd be great. So

this patient is fairly highly unlikely ever to recover self-aware consciousness, is it justifiable to do consider keeping her in the ICU bed and consider giving compassionate care when we know that beds are very precious. Do you think it's compassionate to move them out of the ICU, how can we manage that compassionately? Does anyone have thoughts like when you have a very objective and practical dilemma and then yet you have to show compassion, which happens all the time in the ER or when we are strapped for time or resources. How do you manage this in a way that is humane.

Speaker 4: Yes, like the surviving sepsis campaign right, like it's quite been commonly used worldwide and followed? So, in that if we see there is a one of the recommendations in the last point, they had said about particular point, deciding to cool off care within 72 hours. So, it is a decent time has been a good practice, it is not evidence based, so like where you feel there is a very poor outcome. If you have available resources could be given the chance, longer good medical practice.

PI: I think a trial of therapy is compassionate. Speaker 4: Ya, and that is a part of compassion also.

PI: Did you notice that the manikin we used was African American, so I zoom in a little bit of a nuance there rather than any thoughts on that.

Speaker 1:

the question about whether racial discrimination has any role to play and the answer is no, seems to be, that's not It's not up for discussion really but others may disagree

PI: but do you think race skin color, gender even socioeconomic status justify any kind of ICU dilemma, handling that differently, it happens I mean we've been accused of that. So, not I'm not saying we as a group of physicians in general,

Speaker 1:

only in the sense of positive discrimination, in the sense of making it very clear that you're not engaging as a doctor in any of those discriminations, in the sense of hostile attitudes or behaviors, directed against whatever the category is.

PI: Yes. Any thoughts on systemic biases, with compassion and how we deliver compassion I know we all intend not to be influenced by biases, but literature is out there to say that there are systemic biases present, and the decisions we make and the way we behave. Any thoughts on how we can tweak it out so that we can teach our trainees to recognize those biases in our decision making.

Speaker 6:

Actually, we just had a round of supervision on our ward. And we were talking about an incident and the incident involved a very experienced and very well settled person in our team and the

question came up what would happen if it would happen to someone else who is not in this position, you, and maybe not very good standing. And that brought me to the thought to the thought that we all have a certain kind of narrative. And so, I think it is necessary to reflect on this, and really to reflect on this, why we communicate and why we talk to patients talk to families. If we do not reflect on this, I think there is a, there might be a bias, which is not which we are not aware of.

PI: Right, I totally agree with you that we should be open to the to the thought that there may be in reflection is a very important tool which we sometimes even in our normalizing practice we don't have time for. We call it soft skills, which is a derogatory term for me. We teach our residents at the clinical aspects are much more important than the soft skills. So that's one thing we can at least open ourselves to Christian, did you notice that there were some gender differences and dynamics going on. Do you want to comment on any of that? I know it's a Pandora's box, but since we're talking about compassion, do our team members as well.

Speaker 2: Well, it seemed to me the patient was female. I mean that's a non sequitur at the attendings male and everybody else's female, but I think a common something like a common denominator in, even in the previous discussion is even being present, you know, that is, it's about taking yourself out of your usual place and putting yourself into a place with the other, it's overcoming the difference between self and other non-compassionate doctors I mean just put it as a caricature, are those that are the experts and they, they know what to do and they know what is right and they give the orders, but the others are the others, and they are either others because they are not experts or because they are not the same gender or because they're not the same race or everything together and many other reasons why they're not the self, and maybe this shift is reframing this being able to leave that behind and connect and relate to some others as if the others the like myself are not different, that maybe for me now seems to be describe a little bit of the essence of compassion. So, gender is a role that accentuates the difference between having compassion, and being not compassionate, in the sense of not being able to identify with the other for whatever reason.

PI: So what you're describing is understanding the other person's perspective, basically standing in their shoes,

Speaker 2: understanding and putting it on the same level as my own, not putting it below or above or somewhere else but relating in a way that I can literally sit down with them, I mean this is very difficult for doctors to do if the doctor says this when they cannot do anything and just, you know, the simple step of being present of just connecting earlier. The doctors you know you read it in the in the in the novels they just swear at the bedside, they didn't do any Herodias, they were just accompanied, they were present and I think that's compassionate,

PI: the famous waiting call the doctor right by this look filters in the date. R

Speaker 1:I guess, absolutely I was just going to say that that even though I suspect most doctors have compassion and empathy for some of their patients, but trying to be aware of the the lacunae in one's own personality, you know, a bit of a psychoanalysis probably helps their self- analysis at least, to realize that, you know, there's some areas where one is less empathetic with

some sorts of people maybe from one's own background, and understanding that and I think that's where this visit bias stuff falls in. So, I think, generally, most doctors would say they are compassionate. And then the question is well in those, in which circumstances is one perhaps less compassionate and why. How can one overcome it? So, these are the practicalities the effort, though the underlying answer to your original question is no, you shouldn't adversely discriminate against your patrons.

PI: So do you think there's a problem with our definition of compassion because the survey that I did, which is now. It showed 95% of the nation's marking themselves very high as compassionate physicians. So, then there's no problem right. But do you think there's a problem with the definition that are we saying that like Dr. G mentioned that compassion can change and be contextual.

Speaker 1:

I don't think it's the definition it's the, it's the self-perception aspect of what actually needs 360 degrees of input about whether one's compassionate or whether they have the best of intentions and ratio about one's personality but others may disagree that's

PI: yes so the next step is talking to family members which is hopefully I'll try and do that next. But how can how can the team navigate disagreements around goals of care, like you saw in this the initial can always have a different point of view, the junior resident had a different point of view, when they, when there's end of life discussions and their strong opinions as Christian mentioned that doctor, it's very hard for doctors to do that to put their perspective below somebody else's perspective, how does one actively teach people to do that. Any thoughts in that have anyone done that actively it's implicit explicitly teaching the trainees to have goals of a discussion, or they just follow you in the room and listening.

Speaker 5: Just yesterday I introduced a couple of residents to the goals of having a good conversation and having a good compassionate conversation. So, it's something I really do explicitly. On the other hand, when you work, it means that you work and you show the people around you, how you do your job. And just to add on what Brandon was saying about empathy and doctors thinking they're empathetic. I think I have seen something that I think I will complete an empathetic person, but to be very honest, we had some problems and I'm sure you will recognize it, with not with the gender problems I think in the N, male, female is, well, it's, it's not completely equal but at least we relate in a very equal way to the other sex, but it's the trans cultural problem. We have had some serious problems, problems with Hindu and Islamic people who have very different view of life care that he has, in a very specific way, they have more difficulties accepting the end of life that our culture has, and that has led to some problems where, even my own empathy. Wasn't really challenged, and that is also a challenge I see in our medical environment that we have more and more people of other cultures, and even the very Orthodox Christians in the N who are well weren't really D people, but they have the same problem as Islamic and Hindu people that they have more difficulties accepting that the life is over and that you accept that the end of life is near.

PI: Right, right, that's no perpetual issue that there are different perspectives, even within the teams. M any input from your point of view, you're very experienced and I see okay,

Speaker 3: very experienced as a stretch perhaps but these things are difficult explicitly teaching is, I think difficult and reconciling differences can be difficult, I do, I do sense a little window of opportunity if you look in the context of the two movies. There were data points presented that suggested an irretrievable condition right so you had a pH that was very low. You had a patient with an evolving problem that clearly was pretty dire. You know I think one place to explore is how you have the clinical conversation to come to some sort of a, if not an agreement at least establishes the boundaries around which that information, defines what the patient's trajectory is likely to be, and then that can help inform a greater discussion with the patient if possible or their surrogates if not,

PI: right. So, focusing on the very objective data might help differentiate that, get rid of the biases that we all come with we all unpack our biases and our decision making, whether it's from our background or from our culture, or our beliefs, or even just our experiences we all have different perspectives for example I work in a unit which is run by surgeons as well. And the perspectives are vastly different. So sometimes it's trying to understand the other person not be anchored in where you are, I suppose. I just have one more question,

Speaker 6: maybe, maybe, if I made one point, I think it's not only a question of perspective is what I feel, working in an ICU in a very busy ICU is that very often, it's not clear that we have a differentiation between actions measures and goals that really leads to, sometimes two very bizarre outcomes. So, what we try what I try to do, being on rounds of, when I talk with younger colleagues, is to try to make clear what is the difference between taking some actions like we have this pH of 6.8 and we can. Well, we can resuscitate and everything, so this is a measure, so we could, we can put the person on life support whatever, but we do not take into account what is the goal. So, this is one very important thing we have to stress and draw for your question.

This should be, in my view, this is part of educating younger colleagues.

Speaker 1:

underlying this it seems to me is the question, what are we trying to achieve for this patient and one of them, of course, is said to prolong the person's life, that's a very fundamental concern in medicine and indeed all around us, but at the same time it's in what circumstances should we stop doing that because it actually doesn't help the patient. A and B because the patient wouldn't want us to do it in these circumstances, and it seems to me that one once when asks relatives who are acting as, assuming that they are proper proxies, what they think will be beneficial, and what they think the person concerned with actually one at least one can find out the opposite of those questions and we find very different answers in different cultures in the broader sense of cultures and some people say to stay alive and to keep them alive and if you've got that perspective, then you have a very different sort of discussion and in the problem, that if actually recognizing that the end of life is now coming about. So, to do it as peacefully as possible. We will have a different sort of discussion and problem. I mean if it'll be obvious, I think you're about to agree with individual stocks of people that agree with it's a problem.

PI: It's very difficult from a family point of view, even if there is, I recently lost my mother and it was clearly an end-of-life thing, but it was very hard as, even though it's a family of physicians, it's very hard to, to accept those, those basic things which are very obvious to physicians. So

understanding the perspective is important but then how far do you go and is that compassion or not is the question. The last question I have, and then statement from everybody before if you want, is that how do you navigate pain issues that was specifically asked by one of my team members that in a terminally ill patient how much analgesia do you deliver and where do you know that the doctrine of double effect where you would give it for pain control and not worry about us at least one has established, cmo status comfort measures such as before you start going down that route of palliation. Do you have any experience or any issues around analgesia at the end of life.

Speaker 5: I can be very honest and can surprise you, in an endless, we give as much as needed. That means if someone is terminally ill, and even if someone is not terminally ill, the real, the main issue is relieving the pain, and it means that if you need to give more morphine you give them a morphine and if, if, fear is a problem that you will well you put a hand to shoulder or you give some benzodiazepine you give whatever is necessary, um, you're never afraid to because too much. You give as much as you need and it means if someone is in pain, and you give two milligrams of morphine per hour, and he still is in pain and you double the dose, they give an extra bonus. And so you go on, just to be sure that the patient is not in pain anymore, so it's not the dose which counts but it's the proportionality of treatment

PI: isn't like a protocol or a checklist that you have that helps you, rather than leaving it to the person's perception.

Speaker 5: Yeah, we have protocols and the protocols are very well very rational. That means that you start on a low dose, and you really observe the physiological consequences of pain and explains that when the patient is still in pain, and then you will give more morphine, until the patient is comfortable. we never have any fear of we give too much, because when you can explain why you did, what you did there is no a problem. And then it doesn't mean that we kill patients because it's always a sort of misunderstanding that we kill people, we never kill people, we are we are convinced that when you give good pain relief, it's even in the benefit, even if, even if you think about life expectancy, someone who is in terrible pain will die earlier than someone who is comfortable and can sleep and can rest,

PI: right, anyone else.

Speaker 2:

I just wanted to quote some data which we collected in the US to study; it's coming up now Lancet respiratory net question. The fascinating thing is, Netherlands actually UK too were booked into Northern Europe, Germany with many patients was booked into Central Europe and we found the difference was that the northern European countries and this confirms what we're going to pick up, because has been saying the treatment limitations were about one to two days earlier, usually, immediate after the first day, patient there were more patients surviving actually being discharged alive from the ICU and from hospital, and there were fewer patients who died under maximal therapy, I mean this is what you want to avoid right except for those, maybe where you go with unlimited trial and you see, but the more patients you have who die despite giving them the maximum therapy, I think, the less patient centered the medicine is that you

perform so it seems there's a kind of evolution of palliative care in the ICU. This is the picture that evolves and these northern European countries like the Netherlands probably like UK and Scandinavian countries are way advanced compared to what we do in Germany, for instance, and even much more than when you go down to Spain and Italy and Greece. And this reflects also, the variety and the global variety between North America, Australia and Asia and even India and Africa.

Speaker 6:

Listen. Also, in Germany. We have the palliative care guidelines and the palliative care guidelines, says that you should titrate medication administration and you should do it by pre prepared plants. So, but there are, there's no window of dosages or something but titration is the demand it is what is demanded. And to do this within maybe predefined ways within an institution. This is what you should do maybe an institution and this is what we do in our institution as well. So, but, again, I would just refer to what I said before, navigate pain. It's, again the question in my view of measures actions and goals. So, what is my goal. And with this in mind, there are little restrictions, maybe notice with restrictions.

Speaker 1:

And I think that your goal is hugely important here and what C said earlier, is well worth reflecting on that is Is your goal to end the patient's life. Or is your goal to reduce the patient's pain and suffering, and that makes a huge in philosophical terms, those are different actions, even if it's exactly the same processes. If your intention is one thing to end the patient's life that's a different action from doing the same thing but with the intention, only of relieving the patient suffering, and there's a test for that. There's a patient seem to have got less suffering in which case you can reduce your activities. If your intention is to reduce the suffering. If your intention is to end the patient life so you don't reduce the, the, the activities until the patient is dead, so there is a test to see what your intention is, and of course for some people, doesn't matter what your intention is it's alright to kill them for good reasons and circumstances for other people that's a red line that you mustn't cross.

Speaker 6: Yes. And this is not also a physical philosophical question this is also a legal question at least in Germany. And this point is stressed very much

Speaker 1: Even in the UK, too.

Speaker 6: And you'd better write down what you're doing and what your intention is and maybe also in a detailed way like you said, what is your goal. The goal should not only be just relieved patient's pain but, however, better, what is the, what do you mean by it, what is the like look at restoration look at stress signals and all these things,

PI: there's something more structured in the protocolized retrospective review as we are in a very litigious society especially here in North America, but I'd like to thank everybody now I know I've taken up one hour of your time. I will be sending an email of thanks as well and I hope that we can make some progress and as I know this discussion can go on forever, and it may result in nothing but it's wonderful hearing your point of view and your perspective. Thank you very much and thank you for your time. I really appreciate it.

Speakers:

Thank you. That was very good, Thank you for this discussion . Thank you very much.
