# Supplementary material for: Drivers and drainers of compassion in intensive care medicine: An empirical study using video vignettes
Source: PLoS One. 2023 Mar 23;18(3):e0283302. doi: 10.1371/journal.pone.0283302 (PMC10035878; doi:10.1371/journal.pone.0283302)
Supplement: S2 Appendix — (DOCX) [file pone.0283302.s003.docx]

# Compassionate Care FGD 25^th^:

**8 Participants Including the PI Conducted through Zoom**

PI: I'm going to start; everybody can the rest of the participants can trickle in as their time allows. Thank you so much for coming today. My name is SS I'm an intensivist and an ethicist and anesthesiologist at B This study that we're doing is IRB approved and we will be recording and that's just for analysis purposes later on. The names will be removed from the analysis. So, it will just be so that we can transcribe it and analyze it with the social scientists. So, as you know that we had a previous phase to the study, which was a survey and with open text answers, and now we're doing a focus group discussion, and hopefully we'll be doing focus groups with family members and patients as well if IRB allows. So, I'm going to just go around and have everyone introduce themselves give their names and just a city or state because we have people from different parts of the world. And also, after that, we're going to show two short movie clips that we have made here at, which kind of break the ice for the conversation that we'll have and then we'll have a discussion with some points which I will interject and then we can see what everyone has to say. So, M, would you like to introduce yourself please?

Speaker 1: good afternoon. I am M I am anesthetist and pediatric intensivist and I worked in T in the pediatric intensive care unit of B, for the last 20 years.

PI: Thank you, Dr. P

Speaker 2: I'm P. I met at S, and I do cardiac medical and surgical ICU and cardiac anesthesia.

PI: Thanks for joining Dr. S

Speaker 3: I am anesthesiologist, intensivist and I do a cardiac ICU surgical ICU and obstetric anesthesia.

PI: Dr T, is that how I pronounce it?

Speaker 4: my name is D T I'm a clinical director of care medicine for mercy clinic based out of S. And my practice is primarily medical ICU.

PI: Okay, good to have you Dr. F

Speaker 5: I am a trauma surgeon in C, and I do general surgery trauma and critical care. PI : Great, thank you. Thanks for joining, A

Speaker 6: I'm working at university medicine graphs in G and I'm an ICU nurse and nursing ethicist.

PI: Yes. Great. Thank you. Thanks for joining. We have C.

Speaker 7: Hi, I'm C I'm a pulmonary intensivist down at C in.

PI: Thank you. Thanks a lot for joining. So, with that, I'll just share my screen and show the two short videos please bear with it. It's not professionally done, although we did it in our simulation center. But I was the director and the director of the unit was actually the acting lead so we'll start with the first one. Which first I have to make sure that I share the sound sorry.

# 1^ST^ Video Playing:

Actor 1: for a vasopressor. Actor 2: It's at 2.4.

Actor 3: I think we need to have surgery or I take a look at her. We got to get the bleeding under control.

Actor 1: Dr. Rogers, she's post cardiac arrest and on for maximum dose basis, Actor 2: and a pH of 6.8 shootout

Actor 3: she needs to go to the OR to control bleeding, Actor 2: her wishes were no heroic measures.

Actor 3: She was not aware of what she was saying the besides, we can't keep her in the ICU, we need the bed. She

Actor 2: should we call her husband, or son at least, and have them come and sit with her. Her stomach is really distended and she's in pain could begin to some morphine.

Actor 3: morphine, it's just going to kill her. We need the bed we need to get her out of here.

PI: Okay, I'm going to share another video soon. So, the second video is a similar scenario but it is a pivot on the way that things are done and the communication that occurs and the behavior of the participants.

# 2^nd^ Video Playing:

Actor 1: original press vasopressin,

Actor 2: vasopressin is at 2.4, and a pH of 6.8. Actor 3: We know what our wishes were?

Actor 2: She doesn't want to have any heroic measures. Actor 3: When we talked with family.

Actor 1: I think we should call her husband.

Actor 2: You know I can get hold of her husband and her son and have them come by. Do you think we could start some morphine, she said, her stomachs distended in a lot of pain?

Actor 3: I think so we have to be really careful about the dosage

Actor 2: certainly, certainly, I do. I can also call on social worker and the palliative care, just to be available as well.

Actor 3: I think that's a good plan and what do you think? Actor 2: I agree I think that's a great plan.

PI: Okay, so, this is a very crudely done and a very naive sort of movie clip. It's supposed to show whether people are embodying compassion in their care and in the care that they gave and in the interpersonal relationships and how they communicate with each other at the bedside of an ICU patient, although it may be far-fetched and a little over the top. This is actually a true scenario and in a previous session that we had yesterday it resonated with people in many ways. So just to break the ice if I could go around get a few comments from people about in the with the lens of compassionate care in the ICU just a few words here and their criticisms or whatever not of the video but of what we're trying to deconstruct what is compassionate care in the ICU. D would you like to start? You're first on my screen?

Speaker 3: Sure, you know, I thought was interesting how in the first video, not only not having compassion to the patient, but also not to the other providers. So, the bedside nurse was really trying to express concerns about the direction of care and it was kind of like those were all set aside and like let's move forward with my plan. And so, I think it's interesting sometimes how compassion is infectious in a positive way or lack of compassion is infectious in a negative way, and it affects kind of the entire team dynamic in the ICU.

PI: Thanks, A Any comments?

Speaker 6: Yeah, I would agree with what has been said but I would like to add that often in the interprofessional team and also towards the patient and the families it's not so much about what but how we communicate what we want to do and what we will do. So it is the way we engage with the people.

PI: Okay, so it's really the communication that comes across which can be interpreted as compassionate or not compassionate. But what if I was saying the same thing as in the first video in a nice way? You know, just my body language was an arrogant and rude like this guy's was and he did it very well. He's not a bad person, but he did it very well. But you know, what, if I was saying the same thing, would that be interpreted as compassionate?

Speaker 6: No, of course not. But I would say it's about leaving the other an option to choose and to say what they think about the situation, what they would do? get to leave the room, or get into interaction and to talk about the values to the things that are relevant

PI: Okay, so not just be fixed mindset and say, well, this is it. Dr. T

Speaker 4: The first thing that I noticed I really couldn't keep my eyes off. Is the guy's cell phone. I think that's a dead ringer for somebody who's not paying attention to what's going on around them. And maybe that's just a pet peeve of mine, but the bond the nonverbal communication that was there, just screened lack of compassion. And I think that's something that is very commonly missed, and I think was well demonstrated in that first video.

PI: Yeah, thanks for picking that up. D our director of the simulation center actually did that on purpose, because he that's a pet peeve for him as well. That distraction and dismissiveness of everything going on around them and just basically arrogance, E, anything to add?

Speaker 5: Um, I guess as from the standpoint of a surgeon, I was just in the first scenario was just thinking, wow, like, this is the part where because I do both sides, right, like I would be asked to come in and provide an opinion. And, you know, it's the way that the patient's been prepped with their families. Sometimes that conversation doesn't go well. So then as the consultant coming in to say, like, we can't have this or not, or be the one to initiate that type of end-of-life conversation. It puts your colleague in a bad spot if you're, you know, going out of your way to ignore a patient's wishes and get other consultants on board and whatnot. But I agree with everything has been said, but it really was really poor. The person that looked like they were in charge was actively dismissive of his colleagues’ opinions and you can tell the nurses trying to help the patient and was being dismissed and that's obviously not good for a team dynamic. And the patients always lose whenever members of the team don't respect each other. So obviously, the second scenario, things are much more collegial and appropriate. Thank you.

PI: Yesterday, somebody said that it's always easier to notice compassion when it's missing. So, the first video made more of an impact on everyone. Everyone knows what's missing. What's needed. But the second one was something that we call soft skills. And when I so my basic

purpose to do this is to teach our trainees. So, when you went in capture, what are you teaching? It's very hard to define. It's very nebulous, abstract thing. So, it's always important to reflect I guess, on how the situations pan out even in our day-to-day rounds in the ICU or just how we, how we behave and carry or conduct ourselves. Dr. F would you like to say anything?

Speaker 7: So, I was taking notes, which I'll throw away because this is confidential because I didn't want to waste anybody's time. I agree with everything that's been said. A lot of learning opportunities in the first video, I think, you know, besides addressing the patient's pain, which I think is also a component of compassion care, I think you guys hit the nail on the head about nonverbal communication. I think when you're when you don't address, you know, the issues in the room, it makes everybody really uncomfortable. And I think that affects trickles down to patient care. Eye contact is so important. Not just to the staff members, but you know, one of the things I've realized when you are sitting with a patient or family really like really connecting with that. It has actually for me, it's a lot of mental energy, but it's so powerful. And I think body language, you know, again, I'm just going to echo everything that y'all said. It's so important. It's so important.

PI: thank you. Thanks for those important tips. Dr. P

Speaker 2: so agree with everything which was said. I thought the second video was really a great training type of video on how people should behave. Relatively straightforward concepts. But the way people behave, the team dynamic was all very positive. The first video probably for me was just so over the top that I mean, you just dislike the person from very beginning it was it was more than just allowing you're not compassionate care that you didn't gets a sense of this was a good position at all. And would if you were going to change it probably have toned it down so that it focused on a lack of compassion, but you still got a sense that this person was committed to, to patient care. You didn't agree with all of that, but he was trying to keep the patient alive and not just, you know, clear out the bed and things like that.

PI: Yeah, I agree. I agree. And we'll come to a few points about that as well about what are the points where compassionate care can be displayed or not displayed and like I said, it doesn't have to be just your behavior or you’re the way you conduct you can be a very nice person, but you may be doing something which is not considered compassionate by other people. I have a few more people and then we'll just go on to the first question, E I think E hasn't spoken.

Speaker 1: Okay. I agree with the others comics in the first video everyone was very impolite. Internally they just wanted to finish the case they didn’t behave professionally, not to the patient not to each other I think and then in the second video, the difference was very obvious. The situation was very much compassionate.

PI: Okay, great. So, in terms of questions, you notice that the patient was not able to, you know, give her own preferences and it was a female patient, it was a patient of color, and that was also done that was nuanced, on purpose because of various other systemic biases that we deal with. But when you have somebody like that in your ICU, and you have, you don't know their preferences and their differences in opinions about goals of care, how do you navigate that in a compassionate way, which is inclusive and also fair to the family and the patient? Any thoughts

on that? Because just as a reference, so I'm on a bereavement group with family members of patients who died during COVID in the ICUs, and 95% of those people and these are all adults, adults children. They feel that the care the doctors or physicians display to their parents was non compassionate. And this is not a scientific study. This is just under because we haven't done any IRB approved studies on family members which are which are very focused on compassion. But if that is a reference, how do you proceed? We all of course, think that we are compassionate and that we do the survey that I did across Europe and the US show that a lot of people majority of the participants thought they were very highly compassionate using a score that is validated. So how do you discuss this disconnect on what we are doing and considering compassionate and what other people think of our actions? Anyone wants to take a shot and has thought about this?

Speaker 2: So I think one of the obvious things for COVID was I'm assuming there was during the time that we did not allow families into the hospital with the patient. One I think for many of them that was viewed as not being compassionate, not being allowed to be with their families.

Without all the outdoors in particular, when they're dying. I think also, the goals of care discussions by zoom are just so completely different than when you're in the same room and many of the aspects which come naturally to us during those talks, body language, etc. You know, you just don't happen to zoom. You can't. You can't get a sense of how the person really feels. And they also are likely much shortened. So, I think this was a lot relate to the fact that the families were really unhappy, you know, my loved one is dying, and I'm not allowed to see them, be with them, etc. And you know, all they're willing to do is have this call. particularly zoom did not work for a lot of people, you couldn't get good connections and all of that . They also have been a lot of anger about that COVID and dying of it , it's different than when people are dying of something that the families understand why they dying and COVID was sort of it occurred, you know that they might recognize it, but wasn't something they expected to have happened beforehand. And some of that anger may have been more or less transferred to the team caring for the patient in the sense that you know, they just don't really care.

PI: Thanks, anyone. Else have opinions about disconnect between what families feel and what we think about our care generally? Maybe not with COVID Yes Dr. T

Speaker 4: One of the things that I have to constantly remind myself of is, you know, our knowledge base and language that we use on a regular basis is so very different than most of our patients and their families. And sometimes you can get some things that are sort of lost in translation as you try to explain very complex concepts of what happens to people in the ICU to families. And that can get things really screwed up because you may feel like you have, you know, simplified the concepts well enough to be able to understand that what that person hears is very different than what you intended. And oftentimes, that you don't capture that in the moment, and it's not until later on that you realize that maybe they don't really understand what it was that you would explain really well and that can really can hurt a relationship that you have with a patient or certain decision makers. And so, trying to focus on, you know, over explaining and making sure that you get some feedback from the person that you're talking to, to make sure that they understand those complex concepts is something that is pretty important that we should all try to do.

PI: David, do you have any methods that you teach your fellows or your residents in the ICU? I know you are a program director that you incorporate, explicitly teaching of compassion based on the scenarios that played out today?

Speaker 3: I do not think that we have curriculum that directly addresses compassion as a concept. I think it's a really interesting one. We do vital talk training with our critical care fellows. And we're in the process of creating that for anesthesia residents. And part of that training, you know, focuses on addressing, you know, the emotional aspect of communication rather than just the data facilitation aspect of communication and so, I think a lot of that, that part of the training is really compassion training in some ways, because it is addressing, you know, addressing the emotion that you see in the patient addressing their concerns, a lot more honestly, than addressing the relay of information.

PI: Right. So, I have a few structured questions that I'll just throw out to the group, please. Do you think compassionate care for distressed relatives should be good that should that be considered when allowing ICU bed occupancy, especially when you have some resource restrictions, when there is not much medical benefit to the patient or it's improbable that the patient will benefit from ICU care but the family's distressed and this comes in everyday in our practice. We just a lot more patients in North America die with intensive care than they do in the rest of the world. And we don't know whether it's just because we have more ICU beds or because we just over medicalize our dying patients. Any thoughts on that? And is that compassionate or because we're getting into family requests or is that is compassionate care, just giving in and letting families have more control in the trajectory of the patient?

Speaker 7: I don't know. I think it's a double-edged sword. You know, when I started first being an attending, I was much more objective about when we have conversations with families. But as time has gone on, I've learned that you know, we have to do things. Well. Not every situation but I think a lot of times we have to do things on the family's processing timeline. Can you hear me?

PI: Yes

Speaker 7: you know, when I, I think a lot of it, is like I think it was D said that, you know, I spend more time addressing the emotional component than I do the actual facts because if the amygdala is all hyped up, the cortex isn't going to be able to hear anything. So you've got to address those emotional components first, or else it sounds like I mean, families are toddlers, but it's kind of like when a toddler is having a breakdown, you know, they're not, they're not going to hear anything that you've said. So, I guess that would be the first comment sort of echo. The second one is I think compassion is also guiding them gently and reassuring them that they're not alone in this process. Oftentimes, I will say to families look at this is a team sport. Part of my job is to check you if part of your job is to check me and if I thought you were making a premature decision because I think a lot of family say if I say DNR and I'm killing my loved one and just sort of try never to take the guilt away entirely, but try and take it off of them to say you know what, you love your husband, wife, whatever so much. Your intentions are good and because

your intentions are good, you cannot make a wrong decision. And I also talk a lot about we want to do things for your loved one. You don't want to do things to your loved one. And everyone kind of has their own lineup. Where do you go from the four to the two? And then I also you know I talk about, I start off by talking about the patient as a person and I say to families, you know, if you had to dumb it down, it's patient values plus medical situation equals plan. And, you know, I'll say to them, tell me what brings meaning to your loved one’s life. And I remember having a conversation two weeks ago, and you know, this wife was having a hard time deciding, and we just kind of forgot that we were in the ICU. And just like, you know, she just loves his dogs and nothing medical and she came up to me afterwards. He was like, that was so helpful.

You helped me put this into complete perspective and help me realize he was never going to get home. And so, I think you know, when you kind of step outside, you know all the statistics and you know, he has a poor prognosis. I think that can really help families in their decision making.

PI: Right Thank you that those are very useful insights. Anyone else on goals of care discussions and how compassion can be explicitly demonstrated in those situations? Otherwise, that's Can I talk about pain? Sorry, anyone? E

Speaker 5: see it. Okay. And I'm standing outside of a building and the trains going by so I apologize if it's loud. But to get back to your original question, because I if I interpreted it correctly, you are talking about does the patient's families emotions and feelings does that should keep us keeping the patients in the ICU longer Is that so? You know, for me like we are, you know, the keeper of the beds and I'll be honest, like, just because a patient's family emotionally wants them to stay in ICU because they perceive they get better care, more compassionate care or whatnot, or some ratios are better like that, in of itself, in my opinion, is probably not a good enough reason to keep someone in that kind of high, high level of care situation. Just because or at least in our institution, there only so many beds and we have to take care of all kinds of patients. But I agree that in talking with patients and families, showing compassion, listening to their concerns and helping them kind of talk through why, maybe their patient or their loved one should be downgraded or they don't need that level of care in the moment. I mean, we can certainly help guide them to understand why we make the decisions that we make. So, I think that's an interesting question, though, because, you know, I think of the day we want to make people happy, right, but sometimes we have to show tough love.

PI: it's very hard to navigate that. I think one of you is a nurse, so I've read some background literature on this, as I was trying to study this and build up the proposal. There's a lot more on compassion in nursing literature, and almost none in medical literature. And of course, social scientists have studied this as well. Do you have any insights from a nursing point of view especially in this scenario, you saw that the nurse kind of acted as a patient advocate in this situation and very often they do in the ICU, and they sort of want to protect their patient from physicians? What is the perspective that Could you summarize that or give me your own insight on that?

Speaker 6: Well, in my opinion, and what I experienced at the ICU is that it's due to the proximity that we have all day long to the patient and also to the family and that as a basis for our nursing care. We need to know so many things about the patient about the social background about if he has a dog or if he has children and what is the main context and so we really, we

develop a close relationship to our patients and it's right what you described that the literature always says we are patient advocate. I don't know if it is really always the case because sometimes no struggle to speak up. Because especially in G, because we have a very strong hierarchy. Also, in the ICU. I don't know what it's like in other countries, but they always say it's better but I don't know, it's not experience. So, I think nurses often have the problem that they have this moral conflict because they cannot act in the way they think that it would be best for the patient.

PI: That's interesting. Well, we better listen to our nurses. Here in America, get in trouble. No but it varies a lot from place to place. So, you're right in saying what you're saying. What do you do you construct compassion differently than physicians and that's why there is a conflict in what you wish you want to say and what you do say.

Speaker 6: I think that's an interesting question? I don't think so. I think most of the physicians and the nurses would agree with the compassionate care means, but we have different in my opinion, we have different goals of care sometimes, because nurses often think about the time after ICU, they think about what quality of life can be achieved. And physicians in my experience often rely to, to bodily, I don't know the blood, all the things they can measure and their short time, goals they can achieve. So yeah, often the problem is that we have different perspectives on what the goals should or can be, which is also a chance to, but there's the conversation missing about these two perspectives. They are not brought together. I think that is the main problem.

PI: Okay. So, I'll move on to the next question. Thank you very much for that. And so you saw that the patient's race was African American, do you think gender, race, socioeconomic status, those are things that you're managing such ICU ethical dilemmas differentially can compassion be? Can this be managed more compassionately? When we're when we're juggling differences in patients of demographics, when we're dealing with them? Is there anything that I know that there's been a lot said about this recently, but systemic biases exist? And how do we explicitly make sure that we are not having unconscious bias when we are treating our patients, for example, just as an example, we had to move patients around during COVID to surge units, which were not traditional ICUs. And because in , a large majority of patients were minority racists who had COVID and were very sick. A lot of these families misunderstood this as you're sending them to a unit which is not an ICU. It was taken to be as sort of a racist action explicitly. And when those patients died, there was a lot of explaining to do by the doctor who was present there at the bedside and the nurse. Then Why was this patient moved out of the ICU? Is it because of their color or their race or their gender, etc., etc. But I mean, those are things which are perceived and those perceptions are important and we are addressing them now and more so than ever before. But does anyone have any thoughts on those gender and race socioeconomic status issues, or experience? Yes, Chris, I

Speaker 7: actually I have to say I'm glad that you brought that up. Honestly, I feel like I need more cultural training. I feel very naive when it comes to knowing about other people's cultures because that impacts how they think how they take information and then how they make decisions. I didn't even notice. Recently; I had a really difficult COVID patient situation. And the daughter was very insistent that she stay with her mother who had COVID and unfortunately, we

couldn't allow for that. And she was ready to leave with her mother. I said, well Your mom will die in the parking lot if you leave. And so, I had a really hard time with that situation. I felt very inept. And we had a cultural consult person come in, and I was so grateful because I learned so much. My main comment is I feel like I need more training to better handle these situations so that I can understand where the patient and family are coming from.

PI: That’s pretty interesting. I've never heard of a cultural, cultural consultant or cultural

Speaker 7: Yeah, it was I didn't know that it existed. And it was basically someone can't remember what country they were from. It was somewhere in the middle east. It was a very specific dialect, a very specific tribe. And I literally spent two to three hours in the emergency department just purely over communication because there were ethical, legal, cultural. I mean, the medical management of this took me five minutes, the rest of everything, and I was trying to be compassionate. Oh, my God, like, you know, this woman who doesn't understand English, we're going to, you know, admit her and then she won't understand anything. And you know, we don't have the right dialect on the translator, iPad. And, you know, I was trying to be compassionate at the same time to say, your safety has to come first and we have to figure out what's more important to you. And I just felt really helpless in that situation. So, I think I would love more training and that sort of thing.

Speaker 5: Um, for my patient population, I mean, I deal mostly with trauma. A lot of people don't choose to have a trauma. And so, we get people of all kinds. I think what helps me and what helps me direct my team if there is a cultural or socio-economic differences, you know, we just keep it about the patient. You know, I like to try to remind people, we're not judges, we're not police, not those types of things. You know, whatever brought that person to the hospital, we still provide same care and if there's a misunderstanding, or we don't understand like, it's okay to ask questions. We are all capable of becoming better people. I love that at your hospital, you'll have a cultural person, you know where I live. in, there's, there's not that much variation. But you know, I trained at Stanford, it was really cool. That there were lots of different types of chocolates, for example, and lots of different, a lot of diversity. And so, I think, you know, recognizing if you have a specific need in your population, having the resources to help you out, but at the end of the day, like he's, you know, if I don't know something, it's okay to ask and it's okay to ask in a respectful way. Because we're all capable of learning from each other becoming better people, but it's, you know, the end of the day, it's about the patient and, you know, what is going on right now? You know, I don't care that this person may have murdered somebody yesterday, or I don't care that this person was CEO of whatever B downstairs like doesn't matter. You know, that's, that's sort of how I view it and, you know, but it's definitely a real struggle. So, I'll have to I'll have to take that idea to my people, the cultural consultant. I like that a lot.

Speaker 2: Yeah, S you really asked to two very different questions. One of them was how we as healthcare providers can make decisions which are not biased. I think that one's actually in concept fairly straightforward, which is to make decisions on objective data, and nothing to do with how we think about the person etc. But to do on the data and possibly to get other people to simply give their opinions on that. The reverse part is much more complex, which is, as you mentioned, distrust when you're not on the same socio economic, racial, ethnic, etc., particularly,

patients who may begin with distrust of physicians, medical care, etc. And that can be challenging, I think. It can take a lot of just discussion, having more people involved particularly if they would be more trusted because they share aspects in common having other family members pastors, etc. involved can sometimes help but it can be can be very difficult and we struggle a lot trying to get trust over time with that comes up often goes to care discussions.

PI: Thanks, And another thing that I learned in my literature search and just general I was reading a lot of history because there's not much in medical literature on this, but there's a lot written in history, and there's the perception that in the 1800s in the UK, for example, physicians were viewed as almost as priests or revered, and there's a famous painting, you may have seen called a doctor, which is hanging at the Tis a physician sitting at the bedside of a dying child who died of typhoid fever. And it was actually the painter’s firstborn son who died and the physician is sitting there immersed in the moment, and displaying something that we now recognize as presence and that's considered, you know, how physicians are revered, and were looked at as somebody who's just present in the moment and sharing the grief and displaying empathy and compassion. And then we fast forward to now where physicians are overwhelmed by EMR and documentation. And, you know, we have no time to sit at the bedside or if we do, it's on our own time. Or, you know, we're multitasking by doing other things at the same time, and it's just not perceived as somebody immersed and sharing their grief or sitting in the ashes as I went to Catholic school. So, sitting in the ashes with someone else. Do you have comments on that? I saw a lot of thumbs up.

Speaker 3: You know, it's interesting, I was talking about that earlier in the conversation around when you're talking about patients, families feeling like we were in competitive, non- compassionate during COVID. I was worried and continue to be worried that, for example, my residence was my partner incredible care, where were our burned outs for during that time and therefore were actually less compassionate or had less time because they were often covering twice as many patients as they would normally and that they could have impacted care. So I think both of those things are real concerns.

Speaker 7: I think it really impacts care I, I am burnout, I am fatigued and I remember patient who was still on High Flow for three weeks, very comfortably. And in the end, you know, it was a 50,60 something year-old next-door neighbor kind of person, and he had decided to make himself DNR. And I remember, you know, in the last 24 hours when I knew he was going to pass, and the wife had made the decision to move towards comfort instead of just aggressive with DNR DNI. The nurses were tearing up, and I wasn't, and that wasn't me and you know, I thought, oh, my gosh, what has happened to me, you know, and I felt really guilty. You know, that I couldn't make that eye contact that I couldn't sit with the family. I did, you know, but not the way I would normally sit with them in their uncomfortableness. And I was grateful later that night when I went home and I cried because then I realized, okay, I'm still human. I still can be compassionate. But yeah, Steve and I completely agree it absolutely our self-compassion, in a way sort of has to come first. Although I'm the worst person to be self-compassionate, work in progress, but I do think you know, provider resilience or compassion, whatever you know, really ties into this, where if we can't, if we're not in the right frame of mind, we're not going to be in front of our patients or families.

PI: So it's a professional mental health challenge for across the board, I think all over the world, not just with COVID I think it was happening. It was just waiting to happen. I wanted to just touch on two important things before I stopped taking so much of your time. When is pain management as you saw in this video clip, analgesia was important? The nurse thought that morphine was required patient was in distress, the physician was hesitant because of various other qualms that they may have. And I see this on a daily basis that analgesia is a real point of contention between team members. And I'm actually starting a study on analgesia at the end of life and looking at disparities with racist. Any thoughts on the double? The doctrine of double effect with morphine? Why are people hesitant to give more and I think in the I think it's two studies coming out in the Lancet very soon yesterday, one of our team members mentioned and that shows that Northern European countries are much better with analgesia at the end of life because they keep giving until the patient is comfortable. Whereas all over the world, even in Germany, and in the US, analgesia is actually less than optimal for dying patients. Any thoughts on that?

Speaker 5: I think a lot of people are just uncomfortable with the actively dying process. I mean, you know, those of us that do critical can we deal with it head on, but I know, for my residents, once we get to that point where we're shifting our focus, I have a lot of residents that are just really uncomfortable with the idea of, I help them but yet I'm hastening things. And I think it takes, you know, for a lot of folks that don't do what we do that that concept is very uncomfortable because at the end of the day, we want to help people. So, I think some of the challenge is just accepting that we still help people even though we change goals. And so, once we identified people that their goal was comfort, or feeling good, you know, we want people to feel good, you know, feeling good is different than like, prolonging life, you know, and so that's something that I have seen in my line of work is that people just are really uncomfortable and me helping them becoming more comfortable with the ideas or the methods of helping patients feeling good. We, we help them get better at pain management and things like that. We also have a really excellent palliative care presence at my hospital. So that that also helps,too

PI: Yeah, that always at C, we used to have mandatory palliative care for everybody who's made DNR any other insights, do you have a different point of view for analgesia where you practice?

Speaker 1: No, I think that the analgesia is the first thing that we have to take care in patients who are terminally ill and I don't know if in other countries. Here in, we do not only use morphine, but also transdermal fentanyl patches, which is more convenient, and they don't need all the time to have a secure IV line. So, it's more convenient for the patient and for the family, not to be dependent on the line to take the drug. And I think this helps, where you don’t have an intravenous line on you, but you got to take the necessary amount of drug at the same time. I think this is important and concerning the differences. You said before on treating patients I think that nurses and doctors are treating the same all the patients no matter their race or economic situation, but that we feel here in G, I think that the families are putting more burden to the doctors into the hospital into the nurses. So, when they return for example, they demand more, they don't accept the situation. On the other hand, when they are refugees or they are minorities, they are more you can deal with them more easily even if you devote the necessary time to talk about the situation, they are more open minded in a way. On the other

hand, I think that the richest families are more stubborn. They think that they have the power to decide and to want more for the patients. But doctors and nurses I think that they behave the same. This is my experience

PI: Dr. P you have so much experience in the ICU.

Speaker 2: Yeah, so I think two very different settings. One is where the patient really is a comfort care patient that everyone has agreed is dying, etc. And I think people who have had some experience, we really don't have much trouble one thing that really helps and I assume everyone does it is to have comfort care order sets. That's makes it easy for the inexperienced person to order it and everyone the nurses know what to do. And that's not that's not a problem. I think the more challenge is the patient who you believe could potentially survive the ICU course and in whom pain management and it's uncommon to happen but no might indeed be effective in not allowing the patient to survive that if you adequately treat the pain you're going to have to go ahead and intubate the patient will be more hypotensive etc. And most the time that's not a big consideration, but there are legitimate times where pain management is in conflict with essentially patient care goals. And I think we can struggle a lot with that we do have fortunately nowadays lots of ways of dealing with pain that gets around it but we occasionally do see those situations and I think people struggle with their true conflicting goals of adequate pain management and comfort versus patient who does want to, to survive and get through that.

PI: Thanks and often their times not often, but there are times when patients change their minds or the families change the goals and then you were down a slippery slope of analgesia, which is not the goal anymore, and things have changed or the week might change and the attending might change and suddenly the goals have changed. That happens a lot in my opinion. Any other last comments, any of the issues? So basically, this is about compassion and it may not be end of life. So, I don't want to just focus on end of life some of the scenarios I gave in the survey were patients who are alive I had a cystic fibrosis child in the ICU, a teenager and that was not an end- of-life issue but it was all about presence and compassion, etc. And they can be different. And the week the different weeks that the patient was in the ICU, the family's experiences were different and their opinions about compassionate care were different. Although the goals remained the same. We did not change the trajectory of the so what I wanted to capture was what was it about the different weeks which differentiated the perception of compassion and you all know in your daily life and your work and your professionalism? What it means but how can we put our finger on it and teach our juniors that this, is it emotional intelligence, is it presence is it being aware of other people's including them in their in your opinion? Any last comments or pearls of wisdom?

Speaker 5: I just I would I would have to say, um, you know, maybe I'm biased. I mean, I did my fellowship at S and I really enjoyed learning how to do patient centered multidisciplinary rounds and you know, including families, including everybody having everyone on the team have their moment to speak. And so, I took that model of rounding with me to my current position and it was really considered sort of, like a new thing and a Nobel thing and I kept that sort of style in place when I do my ICU rounds and the subjective feedback has been fantastic.

Nurses love it everyone just likes feel included, and nationally I think it helps promote mutual respect amongst other members of the team. And then you know, as much as I can, involving

patients and their families and giving them their moment to ask questions. Um, you know, it was kind of it became like the regular thing when I was a fellow so it was my regular thing as an attending and it's gotten at least for my institution, it's, it's, it's had really good feedback and, as the team's happy and we work together well, I think that helps us, work together better, and be more emotionally full. And I think the compassionate part will be able more easily done.

Speaker 2: Yeah, a comment I'd make on that is that what we really want to do not is not necessarily teach people to be compassionate. We want to teach them to act compassionately. And those are skills that can be modeled they can be taught, hopefully everyone who comes into critical care is compassionate the way they are or they're not what we really want from them is to learn how to demonstrate compassion.

PI: Absolutely. So, with that, I think I've reached the end of my comments on questions. And thank you so much for your insights. I know that it's a broad topic, we can talk about it all day long. And it may not be very teachable, as you said, as they say in the Confucianism. The student when the student is ready, the teacher will appear. So, you don't have to have somebody who was willing to learn it and imbibe it and there's some people who just you can't. So, I think thank you very much for your time and I will be sending around a small gift as well. So thanks a lot, by email, so please look out for that. I really appreciate all your time. Thank you.
