# Supplementary material for: Drivers and drainers of compassion in intensive care medicine: An empirical study using video vignettes
Source: PLoS One. 2023 Mar 23;18(3):e0283302. doi: 10.1371/journal.pone.0283302 (PMC10035878; doi:10.1371/journal.pone.0283302)
Supplement: S3 Appendix — (DOCX) [file pone.0283302.s004.docx]

PI: I am going to record and the reason is I want to analyze it later. It’s not for publication. It’s just for analysis, so I don’t have to take notes while you guys are talking. I know you're driving . You can hear it and it's pertinent. So, I'll just show you the videos.

Speaker 1: a shout out just for housekeeping. We immediately ask, is that something I need to take notes?

PI: No, no, I'm just going to ask you how you feel this whole thing is about your feelings. Thank you.

1^ST^ Video Playing:

Actor 1: for a vasopressor. Actor 2: It's at 2.4.

Actor 3: I think we need to have surgery or I take a look at her. We got to get the bleeding under control.

Actor 1: Dr. Rogers, she's post cardiac arrest and on for maximum dose basis, Actor 2: and a pH of 6.8 shootout

Actor 3: she needs to go to the OR to control bleeding, Actor 2: her wishes were no heroic measures.

Actor 3: She was not aware of what she was saying, besides, we can't keep her in the ICU, we need the bed.

Actor 2: should we call her husband, or son at least, and have them come and sit with her. Her stomach is really distended and she's in pain could begin to some morphine.

Actor 3: morphine, it's just going to kill her. We need the bed we need to get her out of here. PI: Okay, I'm going to share another video now.

2^nd^ Video Playing:

Actor 1: original press vasopressin,

Actor 2: vasopressin is at 2.4, and a pH of 6.8. Actor 3: We know what our wishes were?

Actor 2: She doesn't want to have any heroic measures.

Actor 3: When we talked with family. Actor 1: I think we should call her husband.

Actor 2: You know I can get hold of her husband and her son and have them come by. Do you think we could start some morphine, she said, Her stomach's distended in a lot of pain.

Actor 3: I think so we have to be really careful about the dosage

Actor 2: certainly, certainly, I do. I can also call on social worker and the palliative care, just to be available as well.

Actor 3: I think that's a good plan and what do you think? Actor 2: I agree I think that's a great plan.

PI: So these two very crude and naive videos were scripted and directed by me. And they're not meant for any, you know, high resolution sort of endeavor. It's just to break the ice in terms of and this was unfortunately a real-life situation. And in our previous I've done four of these focus group sessions before this, and unfortunately, it sorts of resonated with different people, different aspects, a lot of aspects and nuances. Even the visuals in this video, including the African American patient, the female nurse and a resident and the body language and the verbal and the nonverbal communication between the attending and his demeanor, behavior, choice of words, all those things are thrown into this small clip. And the reason I did all of that is because we see different variations of this every day in our normal icy lives. And the study basically the research question is, is there IC? Is there compassionate care in the ICU or not? And how can we teach this to our trainees? Maybe we should just go around and introduce ourselves. If you don't mind. D would you like to just quickly say where you are who you are?

Speaker 2: Sorry. Thank you, S for inviting me to this and nice to meet you all. I'm professor of anesthesiology and critical care at and I do neurosurgical and urology care I do in our cancer hospital. huntsmen ICU staff, that one I do, I have done whatever it is my I was on the civic with a lot of LVAD and ECMO patients and I phased out of our surgical ICU with the trauma patients as a level one trauma center and University of U. By just a little bit of a one- line background is it covers about 1/6 of the landmass of the United States. That means we have patients in that come from from reservation from. They come from places they don't have you know the care so we get quite a bit of referrals of patients in dire situations. From you know, quite some landmass in the United States. So very diverse. patient population in the ICU is in a lot of trouble communicating well from an ICU standpoint of families.

PI: Thank you. Thanks for joining. J would you like to say something about yourself?

Speaker 3: Sure. I'm currently in University of C. I've came from right it primarily CVICU. So yeah, ECMO and beds and all of that. But I came to University when I became a level one trauma centers, we've had some growing pains and primarily in the surgical ICU, which is mostly trauma patients and burn ICU. So mostly dealing with the burning the growing pains of all of that

PI: Thanks for joining, K

Speaker 4: I'm at G in central Pennsylvania, which is why the electricity and Wi Fi situation can be sketchy because it's a rural community. But campus that I'm on is in D and it's our tertiary care referral center for the whole. So, we have about 90 ICU beds on our campus. Six different subspecialty ICUs. At midnight tonight, I'm no longer the co- director of all that I'm going back as program director for that group of care fellowship. But similar to D as you know, some of the issues we deal with is we have people coming from three, four hours to get here. Not all of the families that we serve, have Wi Fi. We have automation mentally. So, with there may not even be telephones in the home. This was all really kind of laid bare with the whole COVID issue and the restriction of visitors because a good portion of our system leadership is in, where there's a lot of resources and people aren't coming necessarily from three or four hours. And the thought was communication can be through cellphones and iPads. And with Wi Fi connection. I'm like, well, when you drive a little bit less of us, you realize you're out in foreign country and in communities that shut electricity and all those things and also even for people that have there's a lot of poverty, I mean, it's actually extremely rural poverty where we are and so there's also the issue of you know, even when someone's dying, can the family actually afford to drive? Do they even have a car that works? Because those are all frequently things that we year that serve as limitations to the family's ability to be at the bedside, even before COVID.

PI: So very important issues. I think, currently, that's as you said, it's been laid bare and require focus. R, can you unmute yourself now?

Speaker 1: Oh, yeah, I'm a general anesthesia and surgical critical care and cardiac critical care at the . So, we do have a very large transplant volume in our surgical ICU and I think a lot of issues as far as communication, compassion and goals of care discussion. It's challenged with our transplant patients. So, we do about, I think 200 liver transplants a year and we do a good number of internal transplants too. So, the amount of investment that the surgeons have and their outlook towards the patient. And what the prognosis is that sometimes it can be like a stark difference between words the intensive is ceases to be and what the surgeon sees it to be and the patients are and their families are so invested with the surgeon and their group and they've gone through this journey together. So sometimes I think going through that barrier can be challenging and we have an excellent communication at the table clinic of which I'm a faculty member there and we have created a communication course for anesthesia members. I mean, it's both for CRNAs and for anesthesiologist and for critical care providers. To communicate with empathy and compassion and collaboration with show. We are currently rolling out that course now with all of our physicians and nurse anesthetist in our anesthesia Institute. So, I'm really happy that that's kind of underway. And I'm really happy to be part of

this group. And thank you, S for the invitation.

PI: You're welcome. Thank you so much for joining. So, K I think you missed the first small video that I made here. And this is also part of the center that we're working with. And it's a center for conscious planet and basically it deals with compassionate care and I'm, I call it kindness research. So, they're a couple of projects that I'm leading. One of them is to try and understand compassionate care. So, I have a few structured questions. K I know you missed the two videos. They were basically a short clip of the same actors in two different scripts. So, the first script showed a patient who's at the end of life is in dire straits post cardiac arrest and she's African American. The attending is in a rush to get her to surgery or IR or some aggressive intervention. The nurse is very much advocating for the patient and she's a female, the resident is a female, and they're both trying to say that, you know, we should call it now and not be aggressive as the patient had wished. And also respect her wishes get in her family for the end- of-life care call in palliative care, but the attending is being aggressive about trying to get her out and he actually does say that they're short of beds and we need her to leave the ICU and get an intervention done. In the second clip, the same situation is flipped around and the whole-body language of the attending is very different. He's more inclusive of the opinions of the nurse and the resident. He shows much more, the way he's standing and talking is more inclusive and compassionate is taking into account what the patient's wishes were. And he wants the family to come in and the nurse kind of facilitates that. So, the situation is not something that we're going to discuss today but just around those things. I wanted to hear your thoughts first about the videos or the situation that as I described, so if we can go around and then I'll come to the structured questions that we have to start with Dr.

Speaker 2: Yeah, because I certainly I have been in both situations and I am preferring to be more in a situation of the inclusion of every team member involved and I personally default very quickly, very easily to social work into palliative care and in palliative care there is a moment that sometimes a palliative care needs to re name itself with palliative care is unfortunate so many times with our surgical teams connected with end-of-life care or withdrawal of care which I don't see like that one can indeed you know graduate from hospice even, but just to facilitate the communication to really go down to the core of what the patient would have wanted. So, I do I did light up seeing the second video and this change of dynamics within were in the first video the hierarchy was clearly defined. The doctor has an idea of this is what we can do but not necessarily in the best interest of the patient, certainly not in the wishes, reflection of the patient. So, I would say that is certainly something that critical care needs to move towards this inclusion of the teams that help us being in social work palliative care, we have one group that was renamed now I came to hospital to support it from quality services. So, it doesn't sound like palliative care. But that is a team that involves all these groups together when it comes to a dire situation that release also the stress of the physician in charge in the ICU or the surgeon in charge to just identify that and make it clear for the family members. I think we have a lot to do that's why the second video certainly led me opens ideas this is this is the right direction. This is where we need to go. The first one is certainly something that we have to evolve from for what's possible in critical care does not mean it needs to be done.

PI: Okay, I'll come back to you for a few things since you have such a vast experience in this. Java, what did your gut feelings and first thoughts off the bat?

Speaker 3: You know, I think watching that video just initially I was extremely aggravated. I think that's exactly the example of what we should not be doing and critical care and just seeing the body language seeing the attending physician on a cell phone. I wasn't sure maybe he was reading a script but you know, he was definitely not engaged.

PI: That was the simulation center and he actually brought up the point that you know, I want to do this it's a pet peeve of mine, that I want to be distracted while I'm talking about something really important to someone's life. So, I thought it was a good little thing to do to throw in.

Speaker 3: Yeah, definitely caught my eye and I was really just all I could think of is how angry I was watching that first scenario and how, you know, I always try to tell my team members, you know, treat everyone as if it's your mother or your child and this is not my mother. So, I don't you know, to be honest, I don't think I even noticed that the patient was African American initially, I think I was just really mad and so yeah, I think aggravated is probably the word I would use to sum up my emotions.

PI: How did you feel about the second one?

Speaker 3: Much better? You know, I think it's a lot more like what I'm used to in terms of communication, at least when I'm on service. I have unfortunately dealt with a lot of surgeons who were like, you know, the young man playing the attending physician there. And then, but it usually doesn't digress like that.

PI: There were a lot of nuances thrown in together but I've had each of these situations with different people.

Speaker 3: No, I have as well. And so, I think I've, over the years at least figured out a way to make it much less like that and just seeing that happen again the way it did. The first year or two in my career was frustrating. It's like oh, no, here we go again. That kind of thing. Yeah.

PI: Did a bit of flashback for all of us. K do you have any thoughts on what I describe? I know you didn't see the video you're still muted K

Speaker 4: Sorry, I was relocating the barking dog. So those scenarios both play out. And I've seen both of them the duration of my career I forgot to mention I have a master's in ethics. This whole end of life, decent communication, you know, patient wishes versus what medicine can actually deliver. has always been very keen interest actually have very difficult interaction last week that I had to deal with where a patient was on multiple pressors more than I personally engage in my practice, but my fellow started a fourth press or overnight and a gentleman who was clearly dying and trying to work with the family around that reality and not really being able to get through to them. But so, in general, my observations of physicians have been, seems to be perhaps maybe at least two different psychological things at play in a lot of doctors. I don't think most doctors realize that these things are going on in their heads a couple years ago in conversation with surgeons and a surgical ICU. When I as the intensivist met the patient kind of looked over the entire clinical course and came to the conclusion this person was dying and

attempted to approach my surgical colleagues about what exactly where they telling the family at this point in time? What were the realistic chances based in medical physiology and lack of response to aggressive ICU care with all the machines and all the drugs and all that good stuff that we use? And the answer I got back I think, actually really kind of laid something open to me and that was we've done so much we can't give up. In, in one very simple phrase, my surgical critical care colleague, I think explained a whole lot of what I had observed so far in doing full time critical care for over two decades. I think the other issue that we face, and I'm not sure how it ties into compassion and compassion or not I think there are a lot of doctors and I think other members of the healthcare team but perhaps doctors more than the nurses and therapists that this whole thing about death is the enemy and that it's somehow reflects on you if your patient dies, and so throwing more and more and more stuff, you know, more drugs, more machines and not sitting down and going through that very difficult series of conversations over a couple of days if you're fortunate enough to have a couple of days to work with to try to help the family process through their five Kubler Ross stages of the fact that a loved one is going to die. Right and I think actually so some of the feedback from the family last week was one of the things that they found very, as a great source of stress to them was kind of the mixed messages they were getting from different members of the team that had taken care of their loved one over the past seven months because this was a person who had initially presented back in November. And had been in another hospital over that period of time. So there's this richness of human psychology, and I don't think enough time is spent. I'm glad to see the improvement in communication skill training. And we have a guy singer have been growing communication skills, difficult communications, communicating upon a potentially failed diagnosis, communication with end of life. We've got patient standardized patients’ simulations in a very robust program. And that's clearly important, but I think there's still a lot of work that needs to be done on what goes on in between the ears of physicians. Like why do some people with the same training can recognize when a patient is no longer responding to what we're doing in the ICU and can then make that next decision to sit down and engage with the patient and family and start to work in anticipatory grief and in a compassionate way. And some other doctors just can't. Because I think that's probably a missing element to this whole issue. That I don't know, it's really been explored very robustly.

PI: And I'm going to come back to you because I have a few thoughts on that R. Would you like to say a few words about what you saw? Or I don't know if we were able to see the videos?

Speaker 1: Yes, I was able to see the videos. So the thing that stands out to me, I think all of us would agree that an ideal conversation would look more like the second video that we saw. And I think the part that is standing out to me that is that they're all it's important. Recognize that I think everybody's intention is good that they all want to do what's best for patients, but the way it is communicated and the way we express our thoughts, I think changes the whole perspective on how that conversation goes and how it is perceived and how people feel after a conversation is said, so I think that's what I saw more in the second video is that all the team members were trying to be more collaborative, and they were trying to acknowledge each other's thoughts, responses, knowledge of the situation and trying to work with each other as a team. I think that's what probably made it feel that it was a more of an ideal team-based approach and a collaborative approach animal compassionate approach to the care that the patient had an addition to all the nuances that you added in there showed I think all of them stood out with, you

know, the surgeon being like the tall towering person and a female nurse and an African American patient. All these are elements that add into the bias that plays into our conversation. So that's, I mean, in short, that's kind of what I felt about the both the videos.

PI: Thank you so much for those comments, all of you. Yeah, it's so basically, we're describing presence and emotional intelligence and or lack thereof. But the question I had now is that, do you think when we don't know the patient's preferences, and I don't want to discuss just end of life cares. I just wanted to discuss more how compassionate care plays out because I know a lot has been my own medical ethics thesis was on end-of-life care in the elderly, but we deal with lack of compassion on a daily basis in various other scenarios as well which may not be a dying patient, which may just be a normal patient, or even lack of compassion with the colleagues. So do you think how we negotiate things with our team members, especially around goals of care? Do you think that is something that can be taught how we can teach compassion in those scenarios to people? Does anyone have thoughts around that? And a lot of you talked about behavior and emotional intelligence how we can teach. I think communication is a bit of a cliched word now because we can I can say the same thing to you. But the same words may have different effects if our relationships are different, or if the way I say it or the manner in which I deliver as Rashmi was saying is different. Any thoughts on that?

Speaker 2: There's certainly I would say, yes, I agree with R100% that a transplant surgeon needs to look at things differently. That's just or they couldn't do what they do. I would say there's no doubt about it or, you know, a cardiac surgeon or you know that there's a different setup or baseline that I would say that makes us different to be able to do different things. And however, to get a little bit of this emotional charge out where I default sometimes do is just at present scores risk scores. If I present a risk score of somebody, you know, being in bad shape, or I see the patient is dying, just what K has described as somebody who's on three or four presses now escalated and you know, things are just not looking good to say that the patient is dying is one thing and then but to convey my trying as on emotional in a way very neutral and fake based for one of my team members, is that I present the risk scores as you know, this is an Apache of that or this is a so far that and this is where this goes and these are the numbers I

didn't create those numbers that is research as evidence based medicine. And I think that's time to communicate that to the family members, and to be on the same page when we round together.

So, I default, in with my approach to the who do I have in front of me what how is this search and made of what's the fabric of the surgeon or the person that I'm dealing with? So, I apply this or that to them? And I would say yes, there is a degree that this can be learned, you know that I can teach my residents and my fellows to say, you know, this is an approach that you can choose as something but I have unfortunate come to the place of you know, we say compassionate, you can also say that it is empathy. Empathy is pretty hard to teach. There are key phrases there are key things but you know, from a developmental standpoint, if it hasn't happened between zero to three, the window closes, and the child will potentially not learn it. It's a frustrating thing and we have a narcissist crisis then we know sort of where this comes from. So you can learn some compassion around what the core of it I would go back and K has the Master's in ethics, but it comes back to an ability to walk in someone's shoes and to you know, feel for someone. And I think that is hard. The only way I think I can teach this is by example of what I do and whether someone takes on this example. I'm not sure if that changes the heart the inside the true

capability to that emotion, but I won't give up in critical care, I do the same and this is what I can do, you know, if I have a patient whose family doesn't have a ride, I've ordered uber before that's all the example that I can set. You know when by searches that the last one to do that. I said I'm going to pay for uber to get that family in, you know, it's like, but I see I cannot expect sometimes I came to that conclusion. I cannot expect that someone sees potential what I see. And I don't see that point of view. You know, I could never take a liver out of someone that plays another liver into someone I couldn't do that. But I think this is when this comes together. How do you can learn some techniques around it and then applying it as the responsible provider to say, you know, let's look at scores and that will be a timeline to say we need to call family we need to bring this in. But the true emotion. I don't want to say not to say doomsday mood here, but I think it's going to be hard to change, but inside how someone feels.

PI: Right, anyone else. I don't want to go around and force people to say things and many questions. Yes, K

Speaker 4: I think that it is possible to teach some elements of compassion empathy, by constantly wrestle with just what your theory already called out. You can teach someone communication skills; you can teach them to say the right thing at the right time. But is it really coming from a place of empathy or compassion can be a different thing? And then when we work with the medical students or residents and fellows, I think there's clearly an element at least in my opinion of what you're born with and what your own internal psychological makeup is as to how amenable you are to subsequent education. Right. And so, I think I don't think you could ever educate the sociopath out of being a sociopath and having empathy. I do think you can take the average human being who has been taught things like you know, when someone's crying or in pain, you should say something comforting or ask them what's wrong. Is there anything you can do to help us those basics that hopefully most kids get in their households or in school, and just awareness of others? And then for people who are already internally wired, you can really achieve some amazing emotional awareness and awareness of others, right? So being in tuned, being in tune with the people on your team being in tune with the patient and their family. I don't think that when I think about the people who in my career struck me as the most amazingly empathic and compassionate there's something internal to them, that is the way they were programmed as they were born and how they were shaped by their experiences in their earlier life. You can undo some of that or redirect some of that, but I don't think you can achieve the highest levels of empathy and compassion unless you've got some internal wiring that allows you to be in that space. I don't know if that makes sense or not.

PI: I'm doing another project on emotional intelligence and we're taking scores. We're using a tool before and after the session. It's very interesting.

Speaker 4: when I worked with the fellows, I've said to them, you know, there's, in my opinion observations, there's two classes of physicians who function as technicians of the science and the data in physicians who are healers and to me the difference between a master of the technology and the data. You can choose to be a great scientist and a great technician of the data and the facts but to become a true healer you have to be willing to enter into that space of compassion and empathy even if it makes you personally uncomfortable. I do think there are some people who find it very uncomfortable at first, but we can help support them with education and role

modeling and debriefing sessions after difficult communications. Whatever reasons those communications are different, but I don't I don't know that. I believe that we could teach every doctor to be empathic and compassionate.

PI: So, I have some more questions but anyone else wants to know, Roshni, you're doing a course and you have any pearls about how goals of care and compassion can be displayed and taught to fellows?

Speaker 1: Yeah, so in addition to our you know, the communication course we do, you know, goals of care discussion training for our critical care fellows for anesthesia, critical care medicine. So, we have standardized patients come in and we have them have the fellows have goals of care discussions with. . And what we do is we videotape it, and we play it back to them sometimes I think I've seen a lot of fellows have this aha moment as to is this what I look like when I'm talking to people and having really tough goals of care discussions with patients and family members? Is this what I'm saying? And this is what it is perceived like so I think, can we make all our trainees perfect? Empaths No, some of them are naturally born empaths exactly like what Durrow and Cara said, is they are just born empaths and others are not. But can we bring them closer to where we want them to be? I think absolutely, yes. I think we can teach them empathy. We can teach them the right things to say and how to frame it. And I have seen trainees initially I see them perform, when I say perform it is in a family meeting and how they speak and how they frame it and after they go through training, how they frame the situation, speak to the surgeon speak to the family members. There is a distinct difference in the way they do it. So, in my mind yes, empathy can be taught, can we change their heart and make them 100%? Empaths? No, I don't think we can. But can we get them closer to what we where we want them to be? I think absolutely yes, I think it can be taught to a certain extent.

PI: So some really interesting statistics I wanted to share with you and then maybe Java can start because she didn't comment on this part. So, when I did this first part of the study, we sent out a survey to members of SCCM and ESICM across the Atlantic, so there were a lot of people who filled it out and there was 95% of people. And these were all ICU physicians and nurses. They scored themselves more than very high as compassionate doctors or clinicians. And when you talk to, I'm also looking at data and I'm also part of a parent bereavement group of people who've lost their parents, especially during COVID. And these are people from the normal walks of life. They overwhelmingly feel that doctors in the ICU are not compassionate. And we have close claims data from ESA as well. And various other not very objective because so far studies have not been done but I plan to do focus group discussions with family members, but there is definitely a disconnect between perception of what physicians feel that they are delivering and what is being translated or received by family members or even patients. Any comments Jarva would you like to say something on that disconnect?

Speaker 3: Sure. I think in some ways, we try to have these family meetings when patients are having bad outcomes. And so, I think a lot of it is really whether or not the family members are open to receiving that communication. So, I think that's where a lot of the disconnect may be coming from. I don't know that we do a good job of trying to have, you know, for example, family meetings or goals of care discussions, when they're not at that point in their care. So, I think that would be the one thing major thing about the disconnect. And then I think as everyone

else has been saying, you know, we can try to teach empathy and, and emotional intelligence, but so much of that really is inherent and whether or not you know, our fellows or colleagues or ourselves even can really read the room so to speak. So, in terms of how to bridge that disconnect. I think a lot of it is a case-by-case basis and our own relationship with each and each individual family member or patient. I think that you know so much of it, you know, I actually really, really struggle the most probably in terms of how do I teach my fellows how to have family meetings and communicate and you know, I try to let them do it and observe and I think videotaping them with actors is a great idea. You know, I try to let them have family meetings on their own and I try to observe but then they don't quite do it the way I want to do it and I struggle on and I jump in and then they never really get the practice and I hope that they learned something from by example, but you know, teaching them that emotional intelligence, this is probably one of the toughest things we do.

PI: Great. I have two points. I know we only have 15 minutes and it's late in the evening. I'm really sorry about that. But I wanted to pick your minds about these two points. One is, you notice that this was a little bit about morphine, and there's always this hesitancy about analgesia at the end of life and we're starting a study here to look at disparities in end-of-life care in terms of analgesia, so any thoughts about you know, the Atticus two trial is just coming out in the Lancet in which they showing that people from Northern you might be interested in northern European countries like Finland etc. are really good. The Netherlands is really good with analgesia at the end of life and it's all protocolized and it's done and no one has moral dilemmas about giving morphine whereas even were far behind in delivering adequate end of life analgesia. Any thoughts on hesitancy about that? What are the struggles that going on like this? This doctor said, you know, morphine will just kill her and I believe that is a that is a thought in many people's minds. Anyone had experience with that?

Speaker 2: Yeah. So yeah, having worked transatlantic I can tell you yes, there is no such thing like a European safe just like there's no US saying it's too big, It's too diverse in its in itself. And yeah, definitely worth a, you know there is no end of life in G history, you cannot actively withdraw care in Germany, that is not an option. So that is sort of my, I went to medical school in H, that’s a background I have had, and then I trained in and S has physician accompanied end of life decision is you know, to set like the Netherlands so I mean, I come from all these different, you know, things that came on me I like medicine around the world, but it is it is quite different, how much culture and social comes in. And just one more thing how different that was for me too is I worked in. I had quite some meetings with the ethics crew there to educate myself on African American approach, or how to approach the patients how I am perceived. So, I think that is a big thing that I had to learn to and I think that learning never ends. What is you know, what do I need for culture? That's why I'm not saying just emotion intelligence. There's some cultural intelligence, what do you need as a cultural context to understand I need that again, here in Utah having native patients indigenous properly a population, that that is so complex, that I have moved myself a little bit as protocols and I want to be patient centered in my care. I really want to look into that context of where does this patient culturally come from? What is this patient's position in the family you know, how are all these things that come in? And therefore, an end-of-life decision is too complex that I would say you can necessarily protocol eyes it

however they are. The stables that need to be in an end of life across culture is an experience of being in confident peace, when you leave and whatever the spiritual whatever the cultural context is, that that should be respected from therefore I would say yes, protocols in in unnecessary to give you that frame and then it is a lifelong learning about who your patient is, and you know the moments that you have as a patient in the famous to figure that out. And that is certainly something that will be you know, will probably go on for me until I retire. I don't think that word is ever done. But I certainly see the work, you know, and that's why I like the internet and that's why I enjoy talking to other you know, physicians from other countries. How do you see this how is this population in the United States as the melting pot of everything, what is so different here and how is how is paying for see for that? What are the labels, what's the judgment on it, the place such a complex role in it? So I think that I tried to stay as open as possible and having a lot of in the back of my head what to what to pull it from and have no hesitation to ask to fill me in being an elder for natives being it you know, somebody with a Somali background or in a Muslim background or whatever, that I have a cultural get to.

PI: K any insights on cultural appropriation and analgesia or without analgesia?

Speaker 4:I think, about over the duration of my career so in medical school and residency I remember they explicitly taught you treat with whatever doses of narcotics are needed to relieve the pain because people don't get addicted. If you're using appropriate dosage right and now the pendulum has swung the other way with the significant death rate associated with our narcotic overdose, whether that's prescribed or bought off the street, and I know at least here in Pennsylvania and within the system I'm working in, we now get epic. Basically, warnings flashes about the dose that we've ordered and how many morphine equivalents it is and what percentage over the daily recommended morphine equivalents per day that this X years old X weight patient X gender patient should be getting right. And so, and then the other thing I think that's had a chilling effect on appropriate end of life, relief of pain and suffering is that clearly there are some people who participated in active euthanasia gave intentionally gave massive doses a narcotic and did not have the patient's consent, but there and they've been prosecuted and they've had prison terms or they were found guilty and they've had prison terms assigned to them. But there have been other people that have been brought up who had to go through that whole process right. And we're found to be innocent, but when you've been through that once in your life, as a provider, you're never going there again. Right. And so like the group that I worked with, although, as far as I know, none of those prosecutions have been in the state of Pennsylvania. I can tell you that a good number of the doctors I work with, and even some of the nurses are aware that the United States and so they're like how do you know as a practitioner, that if you do the right thing for the patient with regard to these narcotics, that you're not going to somehow find yourself being scooped up by the local sheriff or police department because the family then goes and claims, you murdered their loved one, because you gave a big dose of narcotic at the end. of life, and then they stopped breathing.

PI: Right? There's a lot of hesitancy and definitely, there's context behind all of these decisions and attitudes. So, we've reached the last five minutes or eight minutes and I'm really grateful for everything you've said. And this discussion can go on all day. So, I'm hoping that there's another stage where I can talk to families, and then come back and talk to all of you again, but as a last closing statement from all of you anything about gender issues, or I wouldn't call it systemic

bias, because there's a lot going on, but these two videos did display how some people can be authoritarian and we've had women who are also in that role as well. We're just not going to be inclusive and not listen to others. But how do you deal with teams like that? And you saw the frustration of the nurse, she was exasperated, and she's clearly very experienced. And she was a natural, I don't have to give her a script. She said she does this every day. And she sees these kinds of personalities every day. So, it was clear that she had been dealing with this again and again and leads to a lot of nursing burnout. R, would you like to take that one first, on how to talk about gender sort of conflicts or friction in decision making and attitudes.

Speaker 1: I think that's it's a really tough one, because, like is evident may not be systematic, but it's so deeply rooted in our system. It's a tough one to handle the one thing that I have noticed, at least in the last two years that I have been more vocal and I call it out. When people display gender biases in their conversation and in their communication, especially at the patient's bedside and in the decision making, be it a nurse or be it a surgeon or an intensivist who's not, you know, communicating well and being respectful towards a woman surgeon. So, it is I think it is it's a deep-rooted problem. I think we are trying our best to handle it and learn and educate people about it. What the one thing that is that I have noticed that has helped it's at least at the very least to call out when things like that happen, and to make sure that everybody is included in the conversation and spoken to in a respectful manner.

PI: J have you been called a nurse by a patient or surgeon before? Not that that is insulting but

Speaker 3: no, definitely. I've definitely been called a nurse. I have even been called medical student all sorts of things.

PI: I wear a hat now it says intensivist and my surgical head

Speaker 3: I mean, I think everyone has a different style of handling it. I think it's just inherited who we are. We're all going to have to deal with whatever biases we have, whether you're male or female, you know, and I know I'm C, I look young and the pandemic has definitely made it interesting for me being C. You know, and especially working on the south side of C with all the trauma patients and everything and other family members. I think there are advantages and disadvantages of being who I am. I think in some ways in terms of dealing with goals of care and empathy. I think in some ways it's actually almost advantageous to be a female. Being young may or may not be helpful. Looking but it's there. I definitely I don't I generally don't address it until it impacts the patient's care. I haven't had too many issues here, UFC. Fortunately, a low is a different story where quite literally every surgeon in the cardiac ICU, he was an older white male, like literally every single one of them. And so, I know that the interactions were quite different when I was unserved as compared to like my six-foot-tall white male colleague. But for the most part, I think being competent really is helpful and I think over time, my relationships with various nurses and other support staff and even my own team and the surgeons, I think experience kind of speaks for itself and people know that I have their patient's best interests at heart and I think, you know, really, family members knowing that you're trying to care for their loved ones. I think it's reasonable and I think we've all had to deal with, you know, systemic biases, regardless of what role we play in life, whether or not it's professional

work, or just in general. It makes us who we are.

PI: Thank you so much. Anyone else about genders in the last two minutes, Karen and Dorothea, you've had years and years of experience I don't look it because I color my hair.

Speaker 4: actually I will say I do think there's been less interaction since I've let my hair go its natural gray about you know, you can't possibly say it's always been an age thing and the old wisdom, the gray hair and the wisdom and connection that tends to be a transcultural kind of viewpoint but like starting in medical school dealing with being treated as if I wasn't competent or didn't know what I was doing because I was female from being physically my physical personal space being violated without my consent from male members of the team, to a male thoracic surgeon and saying, Oh, wow, really, you know, she really knows her anatomy and I'm like, why wouldn't I went to medical school, right? Up to up to the gender bias from patients that we serve and from the families and again, I am completing a two-year run as co-director of critical care at my hospital. Well, that was through the COVID era. And we live in central Pennsylvania, which has a whole lot of really interesting worldviews. The majority of which I do not subscribe to, I'll leave it at that. Right but I mean, the first six weeks I started working at Geisinger, I was told by a very senior very woman in medicine supportive man that I work at a very misogynist system. Right. And then separate from that is the cultural expectations community, which is kind of like the 1950s Ozzie and Harriet nature of male female relationships in a lot of households around here and so and then even pass that like with the Amish and Mennonite where women are silent, right, but communities are led by males and in decisions about what kind of expensive aggressive care like transplants for instance, you know, the Amish and the Mennonites tend to shun transplants even if they're thought to be likely to be very successful because the community pays out of pocket, they self-insure, right? So, for a community that operates at a level of autonomy, as determined by the community, elders, not individual people. frequently you'll get a no answer. And but it's the men right and I've actually had families Amish families where they usually the communication goes is I figure out who the Power Broker oldest female was because it's always the oldest female the matriarchal, head of the domestic part of the family's life. And then she takes it back to the elders, and they do their thing, and then she brings their decision back to me, I never communicate directly with the man. So, you know, you could perceive that as extremely biased or you can perceive that as you've got to figure out how to work within the space people are in when you meet them in a very stressful and very painful part. of their life. I think the other thing about you know, I think one of the things about American culture,this idea of autonomy is always being solely in an individually individual person and what I've always called the Marlboro Man conception of autonomy and the rugged and stand by yourself. Don't show any fear of pain, make all the hard decisions and own everything in that decision making chain doesn't really play out with the way most human beings approach difficult decisions or painful, potentially painful conversations. I think the third thing I throw into this whole thing about compassion is work compression for the providers, right?

We've mentioned how for nurses having an obnoxious physician who is not welcoming of your input as a nurse leads to nursing burnout but with the increasing pressure to be economically efficient, in work efficient, and constant focus on RV use. Compassion is not efficient. Right to be to be compassionate and empathic means you're taking a lot of time to learn a lot of detail about people you've just met in the past hours or maybe a day or two ago, in order to gather all that information, you need in order to understand their cultural context or their theological

perspective, spiritual perspective. What overlays of bias that they may have whatever release bias, you have when those quiet reflective moments where you go back and you're like, Okay, so what am I bringing to this interaction that may help or may hinder, creating that therapeutic relationship in which these compassionate or lacking compassion conversations occur without a doubt, again, over this past 18 months with COVID, and we did not get hit hard? So, I'm going to say that up front, my system did not get hit hard. We had to open some ICU platforms that we don't really use because for about six weeks, we were above our capacity of our standard ICU footprint across the system. But when you're constantly running to catch up in your day when you don't have adequate staffing when you when you're, for us in our rural part of the state, we are about 50% of our ICU nurses’ physicians are staffed by full time Health System employing nurses of that remaining 50% cap about 30% of those positions are filled by travelers and the other 20% are chronically unfilled. So, the nurses are constantly working triples, or extremely short. We're short on doctors were short on critical care, practitioners right now respiratory therapy and my system has constantly struggled to fill in large part because we aren't rural.

Nobody thinks living in central Pennsylvania among these beautiful farms and you know, low good real estate value, overall low cost of living low crime. It's hard to recruit people so it's hard. You know, we're human beings, right. And so, I've seen a lot of my colleagues in the 10 years I've been here. In the last couple years there's definitely been a struggle to remain compassionate when you are running around with the hair on fire all the time, whether you're a nurse or a therapist or a doctor in the intensive care unit.

PI: Thank you so much, everyone for your time. And I'd love to talk more about this. But I know it’s six o'clock and you guys have family so that you go. Thanks again, I have a small present for you, which you received by email. It's very small, but just a token of appreciation for your time. And once I analyze all this, I'll get back to you with what we have so that you're in the loop. So thank you so much for all your input and thanks to my friends who helped me out by putting their hands up for their participation. Take care
